# QSAR Studies, Molecular Docking, Molecular Dynamics, Synthesis, and Biological Evaluation of Novel Quinolinone-Based Thiosemicarbazones against *Mycobacterium tuberculosis*

**DOI:** 10.3390/antibiotics12010061

**Published:** 2022-12-29

**Authors:** Jhesua Valencia, Vivian Rubio, Gloria Puerto, Luisa Vasquez, Anthony Bernal, José R. Mora, Sebastian A. Cuesta, José Luis Paz, Braulio Insuasty, Rodrigo Abonia, Jairo Quiroga, Alberto Insuasty, Andres Coneo, Oscar Vidal, Edgar Márquez, Daniel Insuasty

**Affiliations:** 1 Grupo de Investigación en Química y Biología, Universidad del Norte, Km 5 vía Puerto Colombia, Barranquilla 081007, Colombia; 2 Grupo de Micobacterias, Red TB Colombia, Dirección de Investigación en Salud Pública, Instituto Nacional de Salud, Bogotá 111321, Colombia; 3 Grupo de Química Computacional y Teórica (QCT-USFQ), Departamento de Ingeniería Química, Universidad San Francisco de Quito, Diego de Robles y Vía Interoceánica, Quito 170157, Ecuador; 4 Department of Chemistry, Manchester Institute of Biotechnology, The University of Manchester, 131 Princess Street, Manchester M1 7DN, UK; 5 Departamento Académico de Química Inorgánica, Facultad de Química e Ingeniería Química, Universidad Nacional Mayor de San Marcos, Cercado de Lima 15081, Peru; 6 Research Group of Heterocyclic Compounds, Department of Chemistry, Universidad del Valle, A. A., Cali 25360, Colombia; 7 Grupo de Investigación en Materiales Funcionales Nanoestructurados, Universidad CESMAG, Pasto 520003, Colombia; 8 Medicine Department, Division of Health Sciences, Universidad del Norte, Barranquilla 081007, Colombia

**Keywords:** *Mycobacterium tuberculosis*, QSAR, docking, quinolinone, thiosemicarbazone, molecular dynamics and cytotoxicity

## Abstract

In this study, a series of novel quinolinone-based thiosemicarbazones were designed in silico and their activities tested in vitro against *Mycobacterium tuberculosis* (*M. tuberculosis*). Quantitative structure-activity relationship (QSAR) studies were performed using quinolinone and thiosemicarbazide as pharmacophoric nuclei; the best model showed statistical parameters of R^2^ = 0.83; F = 47.96; s = 0.31, and was validated by several different methods. The van der Waals volume, electron density, and electronegativity model results suggested a pivotal role in antituberculosis (anti-TB) activity. Subsequently, from this model a new series of quinolinone-thiosemicarbazone **11a**–**e** was designed and docked against two tuberculosis protein targets: enoyl-acyl carrier protein reductase (InhA) and decaprenylphosphoryl-*β*-*D*-ribose-2’-oxidase (DprE1). Molecular dynamics simulation over 200 ns showed a binding energy of −71.3 to −12.7 Kcal/mol, suggesting likely inhibition. In vitro antimycobacterial activity of quinolinone-thiosemicarbazone for **11a**–**e** was evaluated against *M. bovis*, *M. tuberculosis* H37Rv, and six different strains of drug-resistant *M. tuberculosis*. All compounds exhibited good to excellent activity against all the families of *M. tuberculosis*. Several of the here synthesized compounds were more effective than the standard drugs (isoniazid, oxafloxacin), **11d** and **11e** being the most active products. The results suggest that these compounds may contribute as lead compounds in the research of new potential antimycobacterial agents.

## 1. Introduction

Tuberculosis (TB), also known as phthisis, is a chronic severe infectious disease caused by *Mycobacterium tuberculosis* that affects humans [1]. TB continues to be a serious global health problem, and before the coronavirus pandemic (COVID-19) it was the main cause of death from an infectious agent, being more deadly than HIV [2]. TB worldwide mortality data show that more than one million people die from this single pathogen annually: TB causes an estimated 10 million new cases and around 1.8 million deaths per year [3]. Moreover, it has been reported that approximately one-quarter of the world population shares the TB infection in a latent phase, representing a high-risk population susceptible to future active TB infection [4]. High morbidity, mortality, and the emergence of new resistant TB strains make this disease a constant source of biomedical research; therefore, a cure for TB has been listed as one of the main goals of the World Health Organization [5,6]. Control of TB in most of the world relies on diagnostics tests such as microscopic-based diagnostics with very low sensitivity and a very old vaccine (Bacille Calmette–Guérin; BCG) and finally, very old therapeutics [7]. Hence, multidrug-resistant (MDR) TB is a growing risk for the population worldwide, with increasingly favorable conditions for the bacteria including the HIV epidemic, other comorbidities such as type 2 diabetes, and low-quality life condition in lower and middle-income countries [5,8].

On the other hand, TB chemotherapy involves first-line oral drugs such as isoniazid, ethambutol, pyrazinamide, and rifampicin for approximately six months; in the first two months of the treatment, all four chemicals are applied, whereas in the last two months, only two antituberculosis (anti-TB) drugs are used: isoniazid and rifampicin. Antibiotic resistance requires a different treatment with second-generation therapeutics [8]. Unfortunately, new TB strains develop resistance to first and second generation [9]. As a result, morbidity and mortality are higher in immunosuppressed HIV patients [10]. Furthermore, the lack of therapeutic adherence due to the long treatment time causes TB-infected patients to abort the treatment, which could increase the resistance of some TB strains [8,11].

The search for new antimicrobial compounds has become a worldwide goal shared by multidisciplinary teams. Recently, bioinformatics teams have focused on compounds designed in silico, thus allowing the discovery of new molecular targets without the experimental laboratory phase. Compound discovery requires investigating the structural features of a compound and its interaction with the cellular pathways that allow it to induce or block a specific human disorder pathway/pathogen. For example, there is a lack of compounds that could deliver efficient results against the TB epidemic; consequently, research and the generation of new chemicals are paramount in the war against TB [11,12]. Novel research could lead to the discovery of several compounds with a high probability of targeting suitable molecules, avoiding the synthesis of inactive compounds and clinical assays, thus reducing the compound discovery time. The different in silico methods, such as quantitative structure-activity relationship (QSAR) and molecular docking, play an essential role in gaining this prior knowledge in compound discovery endeavors [13,14].

In recent years, several compounds have been designed based on cheminformatic tools such as QSAR (quantitative structure-activity relationship) and molecular docking [15,16]. Moreover, additional works have proved that employing these techniques enhances the probability of finding new anti-TB structures, with more probability of success than using only chemical intuition, and further reducing investigation time and costs [17].

**Figure 1:**
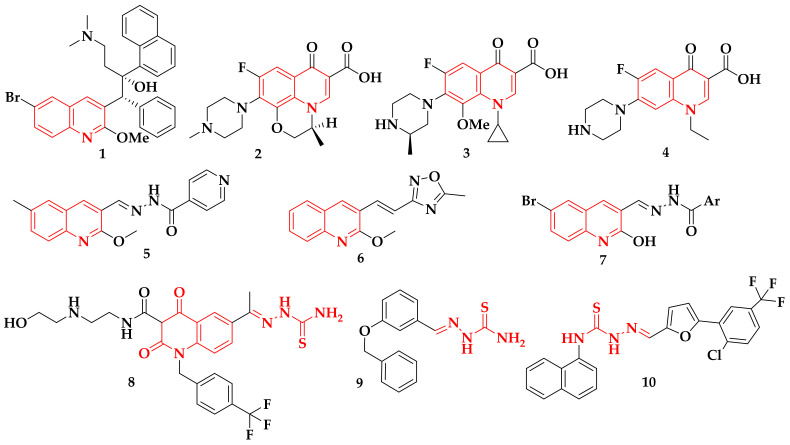
Structure of quinolinone-based anti-TB drugs: quinolines, quinolones, and thiosemicarbazones, used as inhibitors of *M. tuberculosis* H37Rv.

Tuberculosis-related literature suggests the *N*-heterocyclic structures are ideal pharmacophoric scaffolds for anti-TB molecules [18,19]. Moreover, the quinolones and their oxo-derivatives are the principal functional group for several drugs used as second-line antibiotics against tuberculosis, among them, bedaquiline **1** [20], ofloxacin **2** [21], gatifloxacin **3** [22], and norfloxacin **4** [23], used for the treatment of MDR-TB, and other structures such as **5** [24], **6** [25], and **7** [26] are reported as promising inhibitors of *M. tuberculosis* H37Rv (Figure 1). Furthermore, it has been shown that the thiosemicarbazide nucleus allows the generation of compounds with a broad pharmacological spectrum; specifically, thiosemicarbazones have various biological characteristics such as antitumor [27], antiprotozoal [28], antiviral, and antibacterial [29], and their anti-TB properties stand out [30]. For example, hybrids such as quinoline–thiosemicarbazones **8** [31], benzaldehyde thiosemicarbazones **9** [32], and thiosemicarbazones **10** [33] have been reported as excellent inhibitors of *M. tuberculosis*.

In addition, different possible tuberculosis protein targets were reported for these structures, representing a real opportunity to design new compounds with other mechanisms of action, which are a cornerstone to fight against tuberculosis resistance to the current antibiotics [9,34,35,36,37,38,39,40].

Considering the exceptional anti-TB activity of the scaffolds mentioned above, it seems attractive to link both structures, quinolinone and thiosemicarbazide moieties, and prove their potential anti-TB activity. In this sense, this research reports the synthesis of new quinolinone–thiosemicarbazone hybrids and their anti-TB activity evaluation, designed using the combination of quantitative structure-activity relationship (QSAR), molecular docking, and molecular dynamic (MD) studies. Furthermore, the mathematical models herein guided the design of five new compounds that were synthesized and exhibited potent anti-TB in vitro activity against *M. tuberculosis*.

## 2. Results and Discussion

### 2.1. QSAR Modeling

The minimum energy structures for molecules depicted in Figure 16 (see Section 3) were calculated using Gaussian 16. The minimum energy structures were confirmed using frequency calculation with no negative frequency for all data. In this regard, molecular descriptors were computed using these minimum energy structures together with the anti-TB activity (pMIC). Several multiparametric models were generated using molecular descriptors as independent variables and pMIC as a dependent variable (response variable). The most significant ones are shown in Equations (1)–(3), respectively.

**pMIC** = 5.579 + 33.968(**11a**) + 2.791(**8a**) + 0.100(**13a**) − 0.121(**75a**) − 0.275(**24**a) − 12.865(**63a**) − 13.927**χ** (1)

r = 0.95, r^2^ = 0.91, F = 36.10, σ = 0.32, Q_LOO_ = 0.83

**pMIC =** 6.048 + 2.877(**8a**) + 0.065(**17a**) − 0.258(**24a**) − 7.041(**63a**) − 18.108**χ** (2)

r = 0.92, r^2^ = 0.86, F = 31.92, σ = 0.31, Q_LOO_ = 0.77

(3) $$\mathrm{pMIC}=6.401+2.877\left(8a\right)-0.228\left(24a\right)-19.793\chi$$

r = 0.91, r^2^ =0.83, F = 47.96, σ = 0.31, Q_LOO_ = 0.78

where: **1a** = P2_B_AB_nCi_2_SS7_A_KA_m-e_MAS; **8a** = SD_B_AB_nCi_2_SS8_LGP [2-4]_v-c_MAS; **13a** = RA_Q_AB_nCi_2_SS8_T_KA_h_MAS; **24a** = K_B_AB_nCi_2_NS4_P_KA_e-c_MAS); **34a** = RA_Q_AB_nCi_2_MP1_P_NSRW_s_MAS; **37a** = P2_B_AB_nCi_2_SS4_X_LGP [1,2,6]_c-p_MAS; **11a** = P2_B_AB_nCi_2_SS5_X_LGP [1;2;6]_c-m_MAS; **60a** = Q3_B_AB_nCi_2_SS1_P_LGP [1]_m-h_MAS; **62a** = I50_F_AB_ nCi_2_SS7_T_LGP [1]_h_MAS; **63a** = Q2_B_AB_nCi_2_SS5_P_LGP [4-6]_v-c_MAS; **75a** = I50_B_AB_nCi_2_SS1_T_LGP [1,2,6]_v-h_MAS; **88a** = Q1_F_AB_nCi_2_SS6_P_ NSRW_c_MAS; **χ** = molecular electronegativity, **r^2^** = correlation coefficient, **F** = Fisher coefficient, **σ** = standard deviation, and **Q_LoO_** = predictability coefficient (leave-one-out cross-validation). 

**Figure 2:**
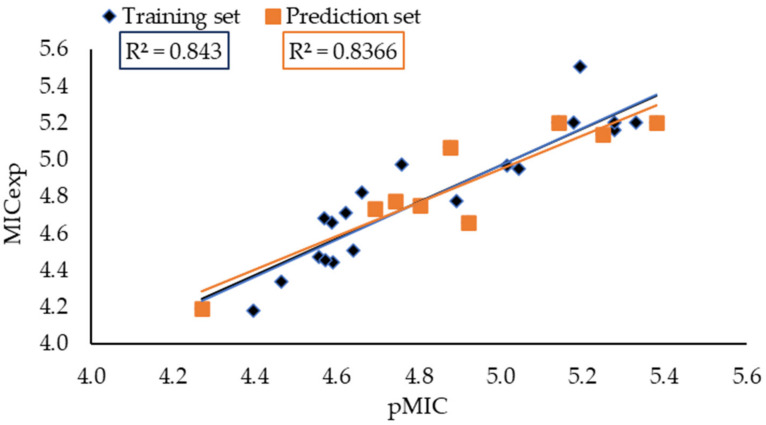
Correlation between experimental and predicted anti-TB activity values.

Data analysis revealed that although all generated models were statistically significant, model 3 presented the best combination between statistics and the number of attributes (only three), allowing better handling and interpretation. Therefore, this model was selected for further analysis and to guide the design of new potential anti-TB compounds. The selected model was used to predict biological activity values for a training set of 23 compounds (71.87%) and a prediction set of 9 compounds (28.21%). Figure 2 shows the excellent correspondence between experimental and predicted values for anti-TB activity.

In addition, Table S1 (in Supplementary File) shows the values of MICexp, pMIC, their residuals, the corresponding value obtained from model 3, and the difference between MICexp and Q_Loo_. A linear trend is observed in both diagrams, indicating an excellent internal prediction of model 3. Moreover, the plot of residues vs MICexp and Q_Loo_ residues vs MICexp demonstrates that residues are uniformly closed to zero, corroborating the absence of consequential errors in the amount obtained. Finally, the Pearson correlation between the attributes for model 3 was calculated and is shown in Table 1. According to these results, there is no collinearity between the molecular descriptors present in Equation (3), demonstrating that the equation is not redundant, with attributes independent of each other [41].

### 2.2. Applicability Domain (AD)

**Figure 3:**
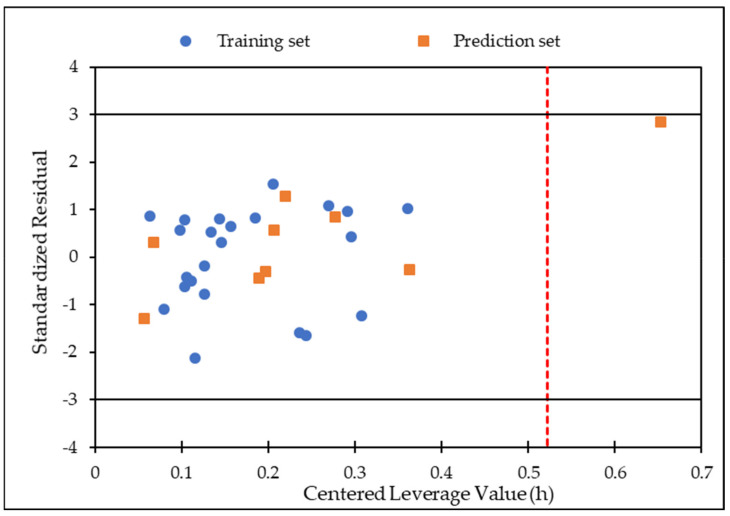
Applicability domain plot of standardized residuals versus centered leverage value of model 3.

The applicability domain analysis was used to detect outliers on the test set and provide reliable and accurate predictions. In this study, the AD was estimated utilizing a William diagram [42]. In this procedure, the leverage threshold (h* = 0.5217) was considered; therefore, a score greater than 0.5217 was used as the criteria to identify if a compound was outside the AD. According to Figure 3, compound **26** fell outside the application domain; therefore, it was removed from the model for future analysis. Thus, according to this AD, the model obtained can be used as a guide for designing hybrids based on quinolines with anti-TB properties since it has a reliable prediction and high confidence [43].

### 2.3. External Validation of the Model QSAR

In Table 2, the different statistical values of the predictive capacity of QSAR model were obtained using the leave-one-out cross-validation (Q_LOO_), leave many out cross-validation (Q_LMO_), Y-randomization test, and bootstrap (Q_boot_). Meanwhile, a r^2^ close to 1 means a good fit for the model; the values of Q_LOO_, Q_LMO_, and Q_boot_ indicate that the obtained model has high predictability since they have a validation value greater than 0.5 [44]. In addition, Y- scrambling values, 0.1367 for a(R^2^) and −0.2774 for a(Q^2^), suggest the statistical model does not correlate by chance.

In addition, Tropsha’s test validates [45] and the accuracy of predicting pMIC values for model 3. This model met all the test requirements, suggesting an accurate prediction (Table 3).

As it can be noted, all of the standard statistical procedures used in this work validate model 3 as a robust, meaningful, and predictable model; therefore, this will operate as a guide for the design of new molecules with potential anti-TB activity. 

### 2.4. Design of the Structures

Model 3 indicates that the anti-TB activity might be related to van der Waals descriptors. The parameter has a positive value in the equation, which means that the more positive this descriptor is, the higher the biological activity. The second coefficient in the equation is related to electron density; this descriptor’s negative value and magnitude suggest a slight decrease in activity as electron density increases. Finally, the last descriptor is molecular electronegativity; the sign and magnitude suggest decreased activity as electronegativity increases. Considering the importance and the kind of descriptors involved in the QSAR model 3, it started from the most active structure (**26**, Figure 16), and some modifications were made to find new potential anti-TB compounds.

**Figure 4:**
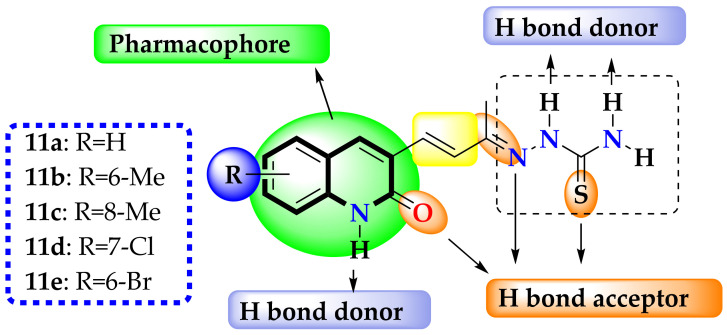
Pharmacophore and QSAR-based designed compounds in this work.

An important factor to take into account is the free rotation and electronegativity of the atoms present in the thiocarbamide group; free rotation increases the molecular volume, which, according to Equation (3), favors biological activity; on the other hand, the presence of N and S makes the electron density and, therefore, the molecular electronegativity, sensitive to the substituents in this molecular region, which, according to Equation (3), would decrease the biological activity. In this sense, the modifications were focused on the quinoline ring. For the structural change, the simplest isosteric substituents reported in the literature were used and are shown in Figure 4 [46]: H, CH_3_, Cl, and Br.

Substitution by methyl groups at positions 6 and 8 slightly decreased the biological activity, while the Cl and Br substituents increased it considerably. This is because the methyl group, which is an electron-donor group, increased the molecular electronegativity and therefore decreased the biological activity. In contrast, the Cl and Br groups, which are electron-withdrawing groups, increased the molecular volume and reduced the molecular electronegativity; therefore, the biological activity was increased in these two compounds (**11d** and **11e**, Table 4).

Table 4 shows the molecular descriptors for the designed compounds according to the selected QSAR model. The values of some parameters used in Lipinski’s [47] rule are also shown. As it can be noted, all of the proposed compounds are potentially active against H37Rv anti-TB strains and meet Lipinski rules.

### 2.5. Molecular Orbitals

In the case of antimicrobial activity, several reports suggest structures with, at least, a frontier orbital located in a specific molecular region displaying a broad spectrum of activity [48,49,50,51,52,53,54,55,56,57], compared to compounds whose frontier orbitals are scattered in the whole structures. This latter characteristic is related to the compound fluorescence ability [58,59,60]. In addition, at least one of the two characteristics pointed out above are presented in the anti-TB compounds already reported [58,61,62,63].

According to Figure S2, the designed compounds could be divided in two kinds: the first one, corresponding to compounds **11b** and **11c,** exhibiting the HOMO (highest energy occupied orbital) and LUMO (unoccupied orbital of lower energy) distributed among all the structure; in this sense, these compounds might act as nucleophilic (hydrogen acceptor) or electrophilic (hydrogen donor) with the biological target by using both quinolinone and thiosemicarbazide parts on the molecule. In contrast, in the second kind, the electron-withdrawing substituents shift the HOMO to the thiosemicarbazone moiety. In line with these results, the designed compounds meet some characteristics in agreement with the already reported anti-TB compounds. Moreover, compounds **11d** and **11e** exhibited the HOMO shape more closely than nitro-substituted 1,3-benzothiazoles (BTZ043), a compound family inhibiting the DprE1 protein. These results confirm that the designed compounds meet the electronic characteristic to become molecules potentially active against *M. tuberculosis.*

### 2.6. Molecular Docking

A molecular docking procedure was carried out to find additional evidence supporting the results obtained from the QSAR method related to the potential antimycobacterial activity of the designed compounds.

In this work, several proteins reported as targets of quinolines and quinolones were evaluated: DNA-gyrase, enoyl-acyl carrier protein reductase, and ATP-synthase [64,65,66,67]; in Table 5 the target with the best scoring values for the designed compounds are shown. The selection criteria were based on the average scoring values for the designed compounds in each protein jointly in comparison with the inhibitor reported for each protein.

The protein decaprenylphosphoryl-β-D-ribose-2’-oxidase (DprE1) was also evaluated, given recent interest in it as a fundamental target for developing new antituberculosis compounds because of the structural similarity between its inhibitors and the compounds designed in this research [68].

Table 5 shows a vina score for the designed compounds against proteins InhA and DprE1, which present the best scoring of the designed compounds. The finding of vina score values lower than 5 suggests the possible formation of complexes between the compounds and the selected proteins. According to the obtained results, the designed compounds present suitable affinity towards both proteins, with compounds **11d** and **11e** having the best performance against both studied proteins.

**Figure 5:**
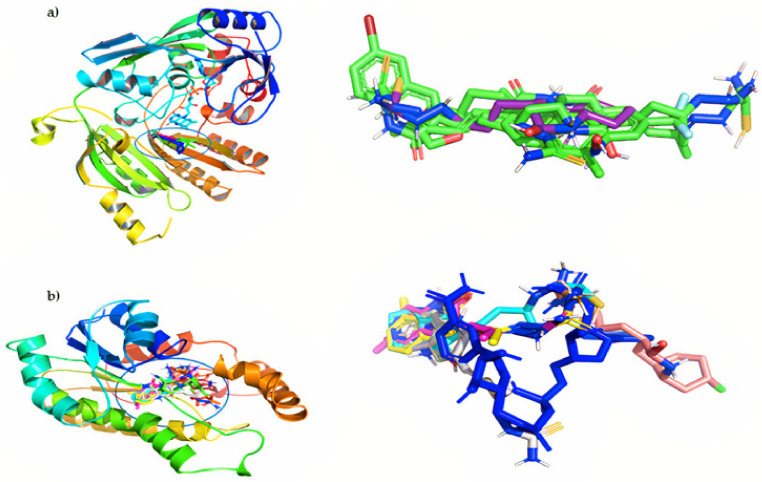
Binding orientation for the most active poses: (**a**) left, DprE1; right, overlap of the studied compounds with the co-crystallized inhibitor (BTZ043, blue); (**b**) left, InhA; right, the overlay of all studied compounds with the co-crystallized inhibitor (INH-NADP, blue).

**Figure 6:**
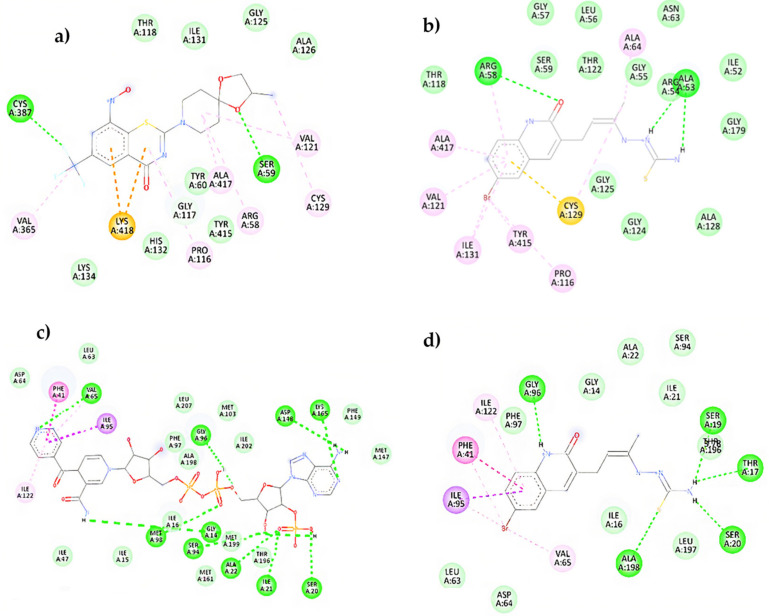
Two-dimensional molecular interaction diagram: (**a**) BTZ043-DprE1 complex; (**b**) **11e**-DprE1; (**c**) INH-NADP-InhA complex; (**d**) **11e**-InhA complex.

As shown in Figure 5, all compounds hit the target at the active site. Furthermore, they overlapped the area of action of the compound reported as an inhibitor in each case, with root mean square deviation (RMSD) values less than 3Å. Finally, to obtain more insight into the role of molecular interaction in the ligand–receptor complexes, the molecular interaction for the co-crystallized protein was compared with the **11e** protein complex obtained in each case. The respective 2D diagrams are shown in Figure 6.

According to Figure 6, compound **11e** interacts with the DprE1 protein analogous to that of the co-crystalized inhibitor (BTZ043). The analysis of molecular interactions in the co-crystallized protein DprE1 suggests that the compound BTZ043 inhibits it mainly by van der Waals interactions where the hydrogen bonds with amino acids Cys387 and Ser59 stand out; these amino acids were reported as fundamental ones in the DprE1 inhibition [69].

In addition, the presence of a π-sulfur interaction (Lys438) can be noted; a strong hydrophobic interaction (Figure 6a). Similarly, in Figure 6b, compound **11e** presents the formation of several van der Waals interactions, such as hydrogen bonds (Arg58, Arg54, and Ala53) and a strong π-sulfur type interaction (Cys129). These results show that compound **11e** presents the same interactions as the co-crystalized inhibitor but forms different residues within the catalytic pocket. On the other hand, Figure 6c,d shows the molecular interactions between the InhA-NADP complex and compound **11e** with the active site of the InhA protein, respectively. It can be noted that the inhibition of this protein by the Inha-NADP complex takes place mainly through dipolar hydrogen bond-type interactions. However, some hydrophobic interactions are essential, such as the formation of π-π bonds and σ-π with amino acids Phe43 and Ile95, respectively.

Interestingly, compound **11e** interacted with the active site of the InhA protein in a similar way to the co-crystallized inhibitor. In this regard, hydrogen bonds with amino acids Ala198, Ser19, Ser20, Thr17, and some significant hydrophobic interactions with amino acids Phe43 (π-π) and Ile95(π-σ), respectively, can be highlighted. In general, compound **11e** showed excellent values in its vina score and molecular interactions similar to InhA and DprE1 inhibitors already reported, suggesting its potential activity against tuberculosis. Finally, it is noteworthy that the 2D interaction diagrams contrast with the molecular orbitals, indicating that for compound **11e**, the thiosemicarbazone fragment has a fundamental role in forming hydrogen bridges. At the same time, the quinolinone moiety would favor hydrophobic interactions with significant energy.

### 2.7. Molecular Dynamics

**Figure 7:**
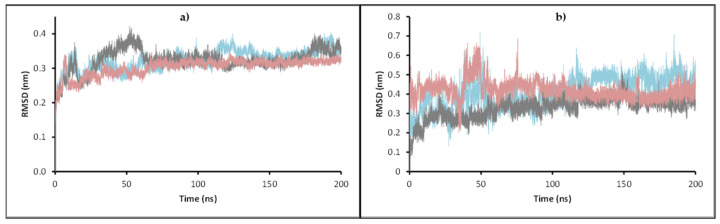
RMSD of enoyl-ACP reductase (**a**) and RMSD of the ligands (**b**) during the 200 ns simulations: **11c** is present in cyan, **11d** in gray, and **11e** in pink.

To evaluate the stability of the complexes formed from the docking calculation and obtain insights into the different interactions that make possible the stability or the lack of it, a 200 ns molecular dynamics simulation was performed for **11c**, **11d,** and **11e** complexes with enoyl-acyl reductase and DprE1 targets. The potential energy, temperature, pressure, volume, and water box size were evaluated to ensure the dynamics ran correctly and reproduced reliable results. For the ligand-enoyl-acyl reductase complexes, the RMSD of the enzyme and the ligands were assessed during 200 ns of simulation (Figure 7).

**Figure 8:**
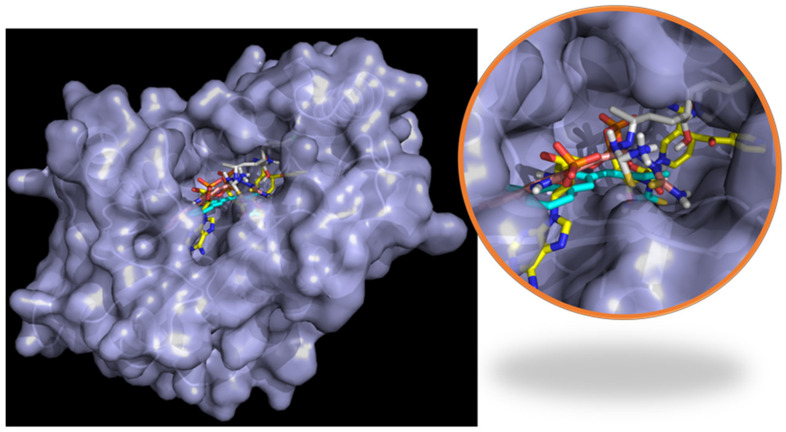
Final pose of **11c** (cyan), **11d** (gray), and **11e** (pink) after the 200 ns compared with (4S)-isonicotinic-acetyl-nicotinamide-adenine dinucleotide (yellow) present in the ligand in enoyl-acyl reductase (blue) crystal structure.

The results show that the enoyl-ACP reductase reached an equilibrium after 60 ns of simulation at a RMSD value of around 0.3 nm for the three systems. This result suggests that the enzyme does not experience significant conformational changes when interacting with the ligands, which may indicate the possibility of positive interaction with them, translating into a potential inhibition of the enzyme. Analyzing the RMSD of the ligands, values below 0.7 nm were found, which supports the reliability of the docking results previously found. Furthermore, low RMSD values also suggest that the complex formed presents a good stability over time, which supports the possible inhibition. A close inspection at the final step over 200 ns simulation showed that the three ligands stayed in the active site, overlaying between them and with the ligand present in the experimental crystal structure (Figure 8).

**Figure 9:**
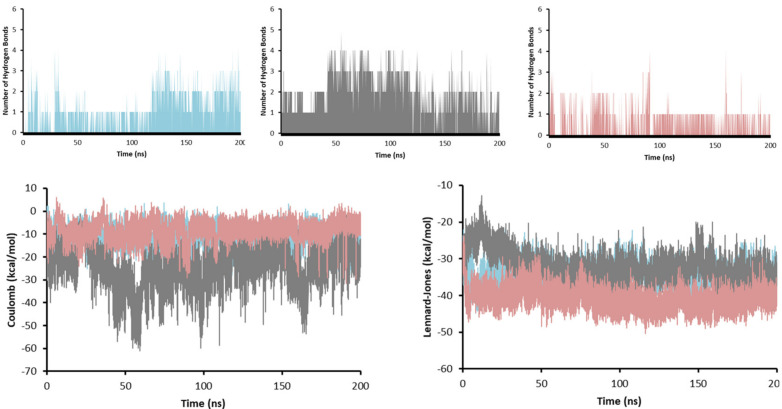
Number of hydrogen bonds, and Coulomb and Lennard-Jones energies **11c** (cyan), **11d** (gray), and **11e** (pink) in complex with InhA during the 200 ns of simulation.

To obtain insights on the strength of the interactions, the number of hydrogen bonds (HBs), and the interaction energies (Coulomb and Lennard-Jones) were obtained for the system during the whole length of the simulation (Figure 9).

The number of HBs is a good indicator of how strong the ligand–receptor interaction is. As more HBs are formed, the more stable the complex will be. In this sense, compound **11d** is the one that may create the most potent interaction with enoyl-acyl reductase, because it formed up to five HBs. Compound **11c** formed up to 4 HBs, 1 HB being the most abundant throughout the simulation and between 2 and 3 HBs in the last 75 ns of simulation (when it reaches equilibrium). For compound **11e**, although it also managed to make 4 HBs, only 1 HB was present during most of the simulation. These results agree with the Coulomb energy profile, where compound **11d** presented the most negative values (around −40 kcal/mol) compared to compounds **11c** and **11e**, which gave an average value of −10 kcal/mol. The Lennard-Jones potential was similar for the three molecules with values between −30 kcal/mol and −40 kcal/mol suggesting the mode of interaction between the three ligands and enoyl-acyl reductase was similar, which is expected for an inhibition behavior.

**Figure 10:**
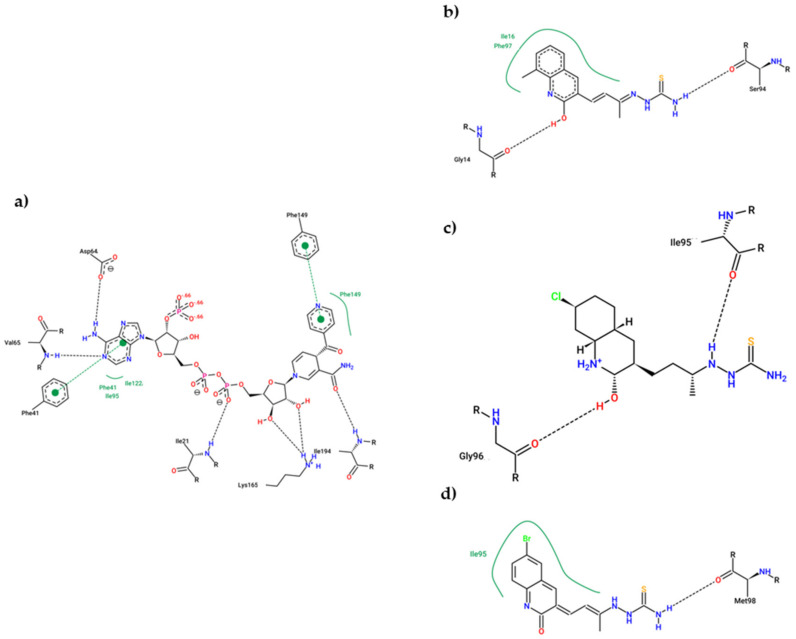
2D Representation of the interaction between enoyl-acyl reductase and (4S)-isonicotinic-acetyl-nicotinamide-adenine dinucleotide (**a**), **11c** (**b**), **11d** (**c**), and **11e** (**d**).

The last point of the simulation was analyzed and compared to the (4S)-isonicotinic-acetyl-nicotinamide-adenine dinucleotide interaction in the experimental crystal structure (Figure 8) to obtain more insight into the interaction between the ligands and the enoyl-ACP reductase. In the co-crystal structure (Figure 10a), the dinucleotide formed five HBs with Ile21, Asp64, Val65, Lys165, and Ile194. Furthermore, it had hydrophobic interactions with Ile95, Ile122, and two π-π interactions with Phe43 and Phe149. On the other hand, compound **11c** (Figure 10b) showed 2 HBs (Gly14, and Ser94) and 2 hydrophobic interactions (Ile16 and Phe97); compound **11d** (Figure 10c) only formed 2 HBs (Ile95, Gly96); and compound **11e** (Figure 10d) 1 HB (Met98) and 1 hydrophobic (Ile95). The dinucleotide managed to form more interactions than the tested ligands, although this was due to the most significant size of the molecule.

On the other hand, similar to enoyl-acyl reductase, the DprE1-ligand complexes were evaluated by employing a molecular dynamics procedure, monitoring the results at a different time, and checking their behavior in the potential energy minimization, water box size, the temperature, the pressure, and the volume of the system during the 200 ns. Therefore, these parameters indicated a correct simulation throughout the calculation time, and the different results obtained were analyzed.

**Figure 11:**
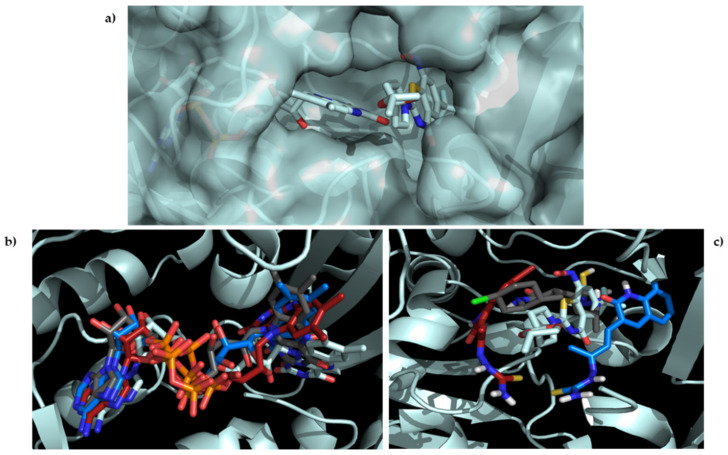
Experimental pose of FAD and BTZ043 in the experimental structure of (**a**) FAD (**b**) and ligands; (**c**) final pose after the 200 ns compared with DprE1. DprE1 and its ligands are presented in pale cyan, **11c** in blue, **11d** in gray, and **11e** in red-pink.

First, the pose after the simulation was assessed to see if the ligands managed to stay in the active site or if the interaction was not strong enough and the ligands left the system. DprE1 complex presents an extensive and solvent-accessible active site pocket that allows the entry of FAD and the ligand (Figure 11a). Interactions in open, active sites are less than those in close pockets, requiring stronger interactions to keep the ligand inside. For the three systems studied, the ligands and FAD managed to stay in the active site after the simulation time, suggesting a strong interaction between them and DprE1. Looking at the final pose of FAD in the three systems, their structure was overlaid with FAD in the experimental form (Figure 11b). This result was expected because cofactors-enzyme complexes are very stable.

Furthermore, due to FAD’s large size, which manages to penetrate deep inside the enzyme, and the multiple functional groups that can quickly form hydrogen bonds with the enzyme, a stable and robust interaction was very likely to be developed, constraining its movement. On the other side, comparing the final pose of the ligand in the active site with the experimental one found in 6HEZ, a good overlay was not found, although they managed to stay inside the pocket (Figure 11c). This behavior was probably related to the lack of interactions between the ligand and the enzyme. Still, the few interactions with the enzyme and possible interactions with the cofactor may be strong enough to keep the ligand in the active site inhibiting the enzyme.

**Figure 12:**
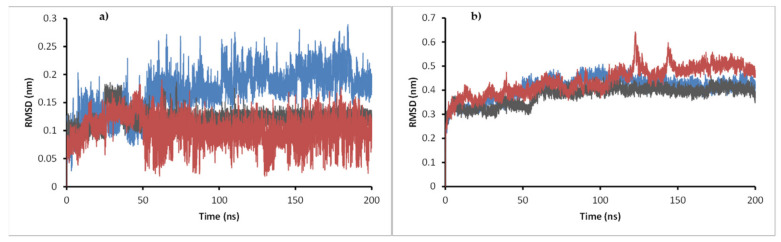
(**a**) RMSD of compounds **11c, 11d, 11e** in complex with DprE1. (**b**) RMSD of the enzyme in each complex during the 200 ns simulations. **11c** is present in blue, **11d** in gray, and **11e** in red.

**Figure 13:**
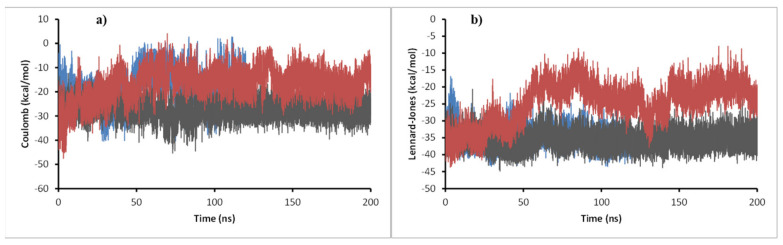
(**a**) Coulomb energy plot and (**b**) Lennard-Jones short-range energies of **11c** (blue), **11d** (gray), and **11e** (red) in complex with DprE1 during the 200 ns of simulation.

RMSD of the ligands (Figure 12) shows that compound **11c** had a significant change in its conformation compared to the result of the docking calculation. Still, values did not reach 0.3 nm in any of the systems, suggesting good stability in the enzyme–ligand complex. For ligands **11d** and **11e**, their RMSD values were less than 0.15 nm during most simulation time. For DprE1 in the three complexes, values were very similar, between 0.3 nm and 0.5 nm, during the simulation. In system **11e**, a peak was observed at 130 ns, suggesting a particular distortion that disappeared in the last 50 ns of simulation where the RMSD kept stable; therefore, the ligands studied do not produce critical conformational changes to the enzyme, indicating a similar binding mode to that of the co-crystallized ligand. In addition, short-range Coulomb and Lennard-Jones energies were obtained throughout the simulation (Figure 13) to complement the stability analysis of the complex studied.

**Figure 14:**
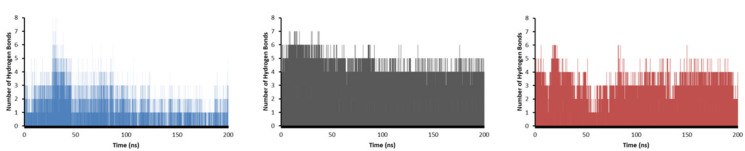
The number of hydrogen bonds for **11c** (blue), **11d** (gray), and **11e** (red) in complex with DprE1 during the 200 ns of simulation.

As shown in Figure 14, all energies were negative, indicating a suitable attractive interaction between ligands and the target. Regarding Coulomb energies, compound **11c’s** energy was 10 kcal/mol lower than that of the other two compounds, suggesting a more robust interaction. For Lennard-Jones potential, **11c** and **11d** presented a similar profile with values around −35 kcal/mol, while **11e** energy stabilized during the second half of the simulation at around −20 kcal/mol.

Finally, a hydrogen bond (HB) analysis was performed on the three systems (Figure 14). The results showed that **11d** was the ligand that formed the more significant number of HBs during most of the simulation, followed by **11e**, and at the end **11c**, which agrees with the most negative values found in Coulomb and Lennard-Jones energies. During the 200 ns simulation, **11d** managed to form 4 HBs throughout the simulation, becoming 5 in more than half the time. Furthermore, **11d** formed 6 and even 7 HBs during a short period. For **11e**, 3 HBs were created during most simulation time, with 6 HBs the highest, but only for a short time. Finally, **11c** formed only 1 HB in the simulation, while 2 and 3 HBs were formed after half of the time. Interestingly, although it was the compound with the least number of HBs during the simulation, at one point of the calculation, it created 8 HBs, which was the highest of all the systems.

**Figure 15:**
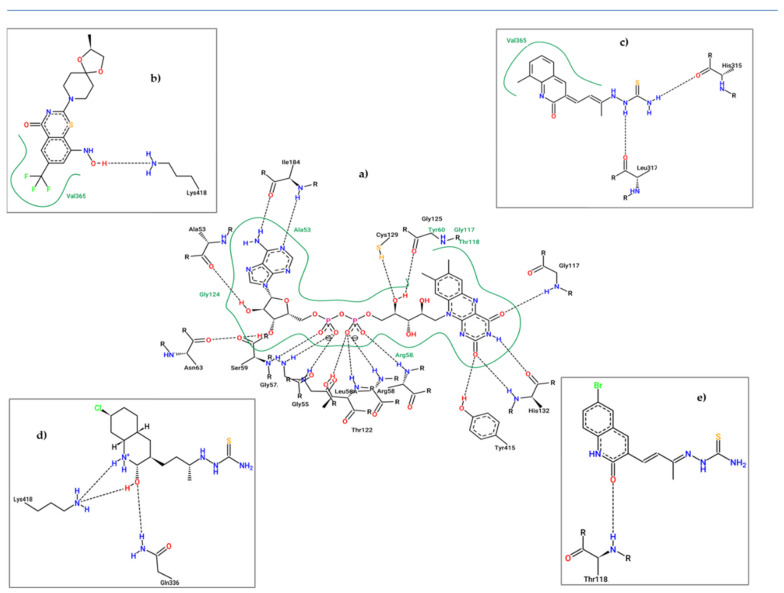
2D Representation of the interaction between DprE1 and FAD (**a**), **BTZ043** (**b**), **11c** (**c**), **11d** (**d**), and **11e** (**e**).

The 2D representation of the final conformation of the complex was analyzed to see which residues were the most important for the interaction (Figure 15). The interactions between FAD and DprE1 were also plotted and HBs with 14 different amino acids were found, explaining the stability of FAD during the simulation. For BTZ043 (experimental), there was one HB with Lys438 and hydrophobic interaction with Val365 where the trifluoro group is located. Compound **11c** presented three interactions; 2 HBs (His315 and Leu317), and 1 hydrophobic interaction with Val365. Compound **11d** showed only 2 HBs; with Gln336 and Lys438, while **11e** had only 1 HB with Thr118. Along with the HBs, any interaction with FAD may also help the stability and inhibit DprE1. Therefore, distances between the different ligands and FAD were measured. Compound **11d** presented the shortest average distance (2.3 Å), followed by **11e** (3.7 Å), and **11c** (7.5 Å). The distance between ligand BTZ043 and FAD for the experimental crystal structure was 4.5 Å.

The ligand–protein interaction was analyzed using the MMPBSA approach (Kumari et al. 2014). In this method, the free binding energies (∆G) are computed considering the van der Waals, electrostatic, and solvent-accessible surface area (SASA) energies (Table 6). According to Table 6, all ∆G values were negative, indicating both favorable and stable interaction between the ligand and the receptor; except for **11d**, the more significant contribution to the ∆G were van der Waals interactions, followed by electrostatic energies, suggesting all of the interactions were driven mainly by dipolar interactions. Interestingly, the complex formation for compounds **11d**-enoyl-ACP reductase was mediated by electrostatic contribution (−77 kcal/mol). Finally, according to Table 6, the compounds interacted more significantly with enoyl-ACP reductase than with DprE1. However, the suitable binding energies (from −12.7 to 71.3 kcal/mol) motivated the synthesis and evaluation of all the designed compounds. The difference in binding energy values obtained for both proteins suggests selectivity in molecular recognition; thus, the compounds designed in this work would have more affinity for the InhA protein than DprE1. On the other hand, although these results are not absolutely indicative of a possible mechanism of action, they do provide additional support for their synthesis and subsequent in vitro testing.

### 2.8. Chemistry

To experimentally validate the results obtained from the computational design, many authors proceed to perform the synthesis of five structures or less [70,71,72,73]. Therefore, Table 7 shows the route employed to prepare the designed compounds using the QSAR procedure, supported by docking and dynamic molecular results. The 3-formyl-2-oxo-quinoline precursors **15a**–**e** were synthesized as presented in Table 7, following the directions of the reported procedures [74]. Two transformations prepared the quinolinone-thiosemicarbazone hybrids **11a**–**e**. The first consisted in the preparation of the chalcone analog derivatives **16a**–**e** by a Claisen–Schmidt condensation between the respective 3-formyl-2-oxo-quinoline **15a**–**e** and an excess of acetone in the presence of 20% NaOH [75]. The mixture was stirred at room temperature for 15 min until all the aldehyde **15** was consumed (monitored by TLC). Then, the reaction mixture was neutralized with AcOH and the precipitate formed was filtered off and washed with a mixture of water/methanol, giving the appropriate chalcone analog derivative **16a**–**e** in 65–88% yields.

The second transformation consisted of preparing thiosemicarbazones **11a**–**e** following the general synthetic procedure (Table 7), where the chalcone analog **16** and thiosemicarbazide in EtOH with the addition of AcOH as the catalyst were heated at 80 °C. The synthesized compounds **11a**–**e** afforded yields ranging from 80 to 92%. The structures of the quinolinone-thiosemicarbazone hybrids **11a**–**e** were characterized by IR, NMR (in DMSO-*d*_6_), and mass spectrometric analysis.

### 2.9. Antimycobacterial Activity

All the newly synthesized compounds were tested against two different acid-fast slow-growing mycobacteria, *M. tuberculosis* H37Rv and *M. bovis* BCG. Isoniazid was used as a reference control, and results were expressed as minimum inhibitory concentration values (MICs, µM) and are presented in Table 8. Antimycobacterial screening showed that all compounds exhibited potent activity against *Mycobacterium* with MIC values in the range 0.03–0.33 µM against *M. bovis* BCG and 0.13–0.17 µM against *M. tuberculosis* H37Rv. All synthetized compounds showed better MIC values than the reference drug isoniazid (MIC = 0.36 µM). Here, we demonstrated the predictive capacity of the QSAR model against the *M. tuberculosis* H37Rv strain. Compounds with highest activity against *M. bovis* BCG were **11d,** with MIC = 0.03 µM, and **11e** with MIC = 0.13 µM (Table 8). Compounds **11a** and **11b** presented an excellent inhibition with MIC = 0.17 and 0.16 µM, respectively; the least active derivative was **11c** with MIC = 0.33 µM. The most active compounds against *M. tuberculosis* H37Rv were **11d**–**e**, with MIC = 0.15 µM and MIC = 0.13 µM, respectively; conversely, the least active compounds were **11a** MIC = 0.17 µM and **11b**–**c** MIC = 0.16 µM.

It is important to note that the experimental values shown in Table 8 (*M. tuberculosis* H37Rv) are in line with the values predicted by model 3, with compounds **11d** and **11e** being the most active. The excellent biological activity of the synthesized compounds is attributed to the incorporation of the thiosemicarbazone system in the quinolinone, since it generates an increase in the molecular volume (parameter **24a**, see Table 4), specifically due to the free rotation of the NH_2_ group (due to its sp^3^ hybridization). Added to the above, N and S atoms provide a decrease in molecular electronegativity (**χ**), which could cause the narrow MIC value among the synthesized products (similar values for each product, see Table 4). These latter results support the hypothesis that the distribution of HOMO in the thiosemicarbazone part seems to be a structural factor related to the antimycobacterial activity.

Complementing this work, the antimycobacterium biological activity for compounds **11a** to **11e** was tested against four genetically different lineages of *M. tuberculosis* multiresistant to isoniazid and rifampicin: a *M. tuberculosis* orphan strain with no match in spolDB4 [76]; *M. tuberculosis Beijing*, an emerging pathogen in several areas and a predominant endemic strain in others that is frequently associated with drug resistance [77]; *M. tuberculosis LAM 9*, a predominant genotypic family belonging to the Latin American and Mediterranean (LAM) lineage [78]; and *M. tuberculosis Haarlem*, whose genotype is ubiquitous worldwide and represents about 25% of the isolates in Europe, Central America, and the Caribbean, suggesting a link with the post-Columbus European colonization [79]. *Haarlem* strains are actively transmitted in urban settings in Colombia [79]. Additionally, two reference strains, *M. tuberculosis* rifampicin resistance strain ATCC 35838 and *M. tuberculosis* isoniazid resistance strain ATCC 35822, were used as internal quality control (Table 9).

All compounds exhibited activity in the qualitative range of good to excellent against the different families of multiresistant *M. tuberculosis,* with MIC values in the range 0.27–66.58 μM (Table 9). Interestingly, many of these compounds showed better activity than the standard drugs (Oxafloxacin, MIC = 2.76 μM). Furthermore, results in Table 9 show that *M. tuberculosis Beijing* was the most susceptible to compounds developed in this work, with MIC values of 0.27–1.75 μM. Noteworthily, compounds **11c**–**e** showed the best activity against most of the tested *M. tuberculosis* multiresistant strains. Compound **11e**, having a bromine substituent on the quinolinone ring, showed the highest activity against *M. tuberculosis* Beijing, being tenfold more potent than Oxafloxacin. On the other hand, compound **11c,** with a methyl group at the 8 position on the quinolinone ring, showed excellent activity against *M. tuberculosis Beijing* and *M. tuberculosis ATCC 35822.* Similarly, compounds **11d** (with chlorine substituent at the 6 position) displayed good growth inhibition against the *M. tuberculosis Beijing* and *M. tuberculosis Haarlem* strain, with MIC values of 0.33 µM and 1.56 µM, respectively.

### 2.10. Cytotoxicity

The cytotoxic activity of compounds **11a**–**e** was determined by an MTT assay on Vero cells (ATCC^®^ CCL-81^TM^) with NaDS as a positive control and dimethylsulfoxide (DMSO) as a negative control. The growth of the Vero cell line was expressed as half maximal inhibitory concentration values (IC_50_). Therefore, the selectivity index (SI) was calculated as the ratio between IC_50_ in Vero cells to the concentration that can kill the mycobacteria. The results are summarized in Table 10, revealing that compounds **11a**–**e** were highly cytotoxic with IC_50_ values in the range of 1.91–3.53 µM. Compounds **11b** and **11e** presented minor toxicity. However, many of these compounds showed a selectivity index of >10 against *M. bovis* BCG and *M. tuberculosis* H37Rv strains, **11b** and **11e** being the most potent, indicating that these compounds selectively inhibit *M tuberculosis* more than normal cells.

In summary, the results obtained in this work suggest that the combination of QSAR and molecular dynamics guided the synthesis of the different compounds with anti-TB activity; moreover, in some compounds, the bioactivity against other strains was more significant than that of the standard compounds. Finally, all the compounds developed in this work showed a selectivity index against *M. tuberculosis* H37Rv >10, with compound **11e** showing the best performance.

## 3. Materials and Methods

### 3.1. Data Collection

The selection criteria were based on compounds bearing quinoline and/or thiosemicarbazone moiety in their structures; furthermore, the guidelines recommended by Alexander Tropsha for the construction of the virtual library were followed [80,81,82,83]. In addition, we calculated the minimum inhibitory concentration of the biological activity using the same protocol. In this work, quinolines are the central nucleus; therefore, a search for antituberculosis quinolines compounds was performed using databases such as SciFinder and PubChem. Subsequently, we searched for thiocarbamides with biological activity against tuberculosis. However, we encountered two obstacles: most of the thiocarbamides were coupled to nuclei other than quinolines outside the application domain. Some thiocarbamides were reported using antituberculosis activities measured in other than IC_50_ or mg/mL.

**Figure 16:**
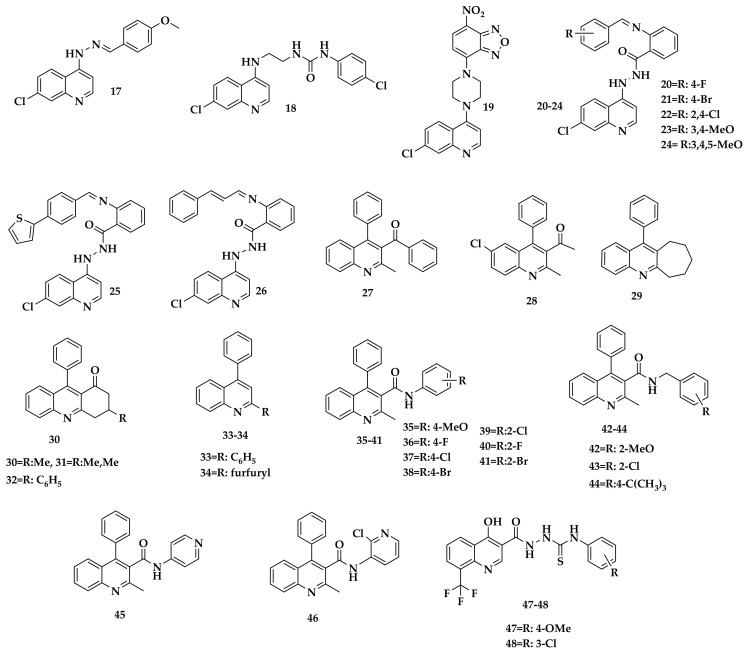
Chemical structures of quinoline-based compounds studied in this work.

Under the criteria explained above, 32 compounds, including three coupled thiocarbamides and quinolines, met the selection criteria. Thirty-two compounds were selected from the bibliography [84,85,86,87,88] and their structures are shown in Figure 16.

It is important to note that the mechanism of action of the selected structures was not taken into account for the construction of the data, since it is not a requirement for the elaboration of QSAR models according to the different research related to this methodology [13,89,90,91,92,93,94,95,96]. Nevertheless, the structural diversity of the compounds studied here, with molecular structures similar to compounds already reported as antituberculosis (fluoroquinolones and quinolones), in addition to the great diversity of isosteric substituents on the pharmacophoric nuclei, guarantee a domain of application suitable for the rational design of new drugs.

### 3.2. Minimum Energy Structures and Molecular Descriptors Calculation

The structures were drawn using Avogadro software (Figure 16) [97], and a minimum energy conformer research was applied. After finding the minimum energy conformers for each structure, these were changed to gjf format and optimized at B3LYP/6–31(d,p) theory level employing Gaussian for Linux [98]. The minimum energy structures were confirmed using frequency calculation. The combination of density functional theory with the set base 6–31G(d,p) was supported by its extraordinary capacity to reproduce experimental results in molecular reaction parameters and QSAR approximations [99,100,101,102,103,104].

### 3.3. Descriptor Calculations

Once the minimum energy structures were assured, three molecular descriptors were computed for the entire data: electronic [105], thermodynamics [106], and topographic [107]. First, electronic descriptors were calculated using conceptual density functional theory [108].

All the electronic molecular descriptors were based on frontier molecular orbitals taken from the output of the optimized structure. Hence, HOMO (highest occupied molecule orbital) and LUMO (lowest unoccupied molecular orbital), were used to calculate other electronic descriptors such as molecular ionization potential (*IP*), electronic affinity (*EA*), electronegativity (*χ*), electronic potential (*µe*), electrophilic index (*ω*), molecular hardness (*η*), molecular softness (*s*), using the following Equations:

(4) $$\left(\chi \right)=\frac{1}{2}\left(PI+AE\right)$$

(5) $$\left(µe\right)=-\chi =-\frac{PI+EA}{2}$$

(6) $$\left(\omega \right)=\frac{\left(µe\right){\;}^{2}\;}{2n}$$

(7) $$\left(\eta \right)=\frac{1}{2}\left(PI-AE\right)$$

(8) $$\left(S\right)=\;\frac{1}{2n}$$

*IP* and *EA* are ionization potential and electronic affinity, respectively; in both cases, parameters were calculated by assuming that the electronic geometry of the cation or anion remained equal to that of the neutral species [109]. Thus, both were calculated employing Equations (9) and (10).

(9) $$EA=E^{-}-E^{0}$$

(10) $$IP=E^{+}-E^{0}$$

Thermodynamics descriptors are those related to Gibbs equations: molar refractivity [109], enthalpy (H), entropy (S), Gibbs energy (G), and molar heat capacity; all of them were calculated for the entire data at 25 °C employing the frequency calculation outputs. On the other hand, the topological molecular descriptors such as accessible area, molecular area, solvent excluded volume, H acceptor atoms, H donor atoms, ovality, partition coefficient, Balaban index, cluster counter, molecular topological index, rotatable number of atoms, polar surface area, radius, attribute form, coefficient form, the sum of degrees, sum of degrees of valence, topological diameter, total connectivity, total connectivity of valence, and Wiener index were calculated employing Chembioffice 18.1 software [109]. Finally, topographic 3D molecular descriptors suggested for cheminformatics studies such as QSAR were calculated using QuBiLs-MIDAS [110]. A total of 3031 3D molecular descriptors were estimated based on many algebraic operations considering linear, bilinear, and quadratic indexes for all molecules in the dataset [110].

### 3.4. QSAR Models

The computed molecular descriptors for the entire data were tabulated and used as the independent variable; the anti-TB activity was used as a dependent variable (pMIC = −log(1/MIC)), where experimental MIC (MICexp) represent the minimum inhibitory concentration (in mol/L). A multiple regression analysis (MRA) was performed using the IBM SPSS Statistics 25 software for Windows to find the most molecular descriptors correlated with the biological activity. Different variable selection methods were used for introduction, stepwise, elimination, backward, and forward selection [110].

In all cases, the obtained models were considered “meaningful” by analyzing the statistic quality; statistical parameters such as the regression coefficient (R), the Fisher value (F), and the standard deviation (s) were computed; as already known, the higher the r^2^ values, the better the model. In the same line, F values correlated variance (r^2^) by the number of degrees of freedom with the unexplained variance (1 − r^2^) by the number of variables in the model. Thus, the higher the percentage of variance explained by the model, the higher the F and, consequently, the better the model. A Pearson correlation was employed to investigate any correlated molecular descriptors in the models. A Pearson’s coefficient higher than 0.7 was considered a “strong association” between two independent variables; consequently, one of the correlated independent variables was ruled out.

### 3.5. Applicability Domain (AD)

For the QSAR building and validation, the applicability domain analysis is a fundamental requirement for determining the model’s reliability and discarding any incorrect dependent variable. It can be defined as the theoretical spatial section limited by the structure, biological activity, and the descriptors selected in the regression model [42,111]. The AD analysis was carried out using a William plot procedure [112].

### 3.6. Validation Procedures

The robustness of the generated models was examined by employing several statistical validation procedures such as the leave-one-out cross-validation and leave-many-out cross-validation methods (Q_LOO_ and Q_LMO_); for the external validation bootstrapping [113] and y-scrambling [114] were employed. Finally, the Tropsha test criterium was applied to the best models [115].

### 3.7. Molecular Docking

Molecular docking is a versatile tool that allows finding minimum energies of protein–ligand interaction structures [116] and has been used as a standard tool for designing and synthesizing new potential anti-TB compounds [69,89,117,118,119,120,121].

This work used molecular docking to test the in silico anti-TB activity of new structures designed by analyzing QSAR models. Two protein targets were used for this goal: enoyl-acyl carrier protein reductase (InhA) and decaprenylphosphoryl-β-D-ribose-2´-oxidase (DprE1). The InhA is an enzyme involved in the biosynthesis of mycolic acid, the major *Mycobacterium* cell wall component [122]; moreover, this is the target of the anti-TB first-line compound isoniazid [123]. On the other hand, decaprenylphosphoryl-β-d-ribose 2’-epimerase (DprE1) is an essential enzyme involved in the formation of lipoarabinomannan arabinogalactan compounds, which are critical to cellular wall synthesis [124]. After selecting the targets, the structures of the proteins were retrieved from the protein data bank webpage (https://www.rcsb.org/ accessed on 1 December 2022) as follows: InhA, PDB ID: 2PR2 and DPrE1, PDB ID: 6HFW. Likewise, their inhibitors, such as (4*S*)-isonicotinic-acetyl-nicotinamide-adenine dinucleotide (INH-NADP complex) and 8-(oxidanylamino)-2-piperidin-1-yl-6-(trifluoromethyl)-1,3-benzothiazin-4-one respectively, were retrieved from the PubChem webpage (https://pubchem.ncbi.nlm.nih.gov/ 1 December 2022) and optimized at B3LYP/6-31(d,p) level of theory.

The structure of proteins with their inhibitors was cleaned by withdrawing any water, inhibitor, and non-native ligands molecules. Then, the proteins cleaned were redocked against their inhibitors to ensure that the docking protocols’ output showed similarities to the protein retrieved from the PDB web page. For this aim, the autodock vina tool was used [125]. The proteins’ PDB ID, active sites, and inhibitors used in the redocking protocols and grillaBox sizes are shown in Table 11.

### 3.8. Molecular Dynamics

Molecular dynamic simulations were carried out to evaluate the different ligand–receptor complexes’ stability over time and to obtain insights into the interactions formed. For the calculation, two enzymes were chosen, decaprenylphosphoryl-β-*D*-ribose 2’-epimerase and enoyl-acyl reductase, while the ligands **11c**, **46d**, and **46e** were used. Furthermore, the most favorable conformation of the ligands in the enzymes resulting from docking calculation was chosen as input for the molecular dynamics simulation. For all the calculations, Gromacs 2019 [126] was employed, AMBER99SB-ILDN [127] force field implemented in Gromacs 2019 was picked to build the topology of the enzymes.

On the other hand, the topology was built using the ACPYPE server [126] and the generalized AMBER force field (GAFF) for the ligands. Before the simulation, the enzyme–ligand complexes were solvated using a three-point water model (TIP3) employing a cube shape and neutralized using chlorine or sodium atoms. Then, the complex was relaxed and equilibrated using constant NVT (number of particles, volume, temperature) and NPT (number of particles, pressure, and temperature) protocols for 100 ps at 300 K [128]. Once the system was equilibrated, the 200 ns simulation was carried out, setting the temperature at 300 K and the pressure to 1 bar [128].

### 3.9. Molecular Orbitals

In the past twenty years, frontier orbitals have played a pivotal role in understanding the molecular interaction between ligand–receptor and a specific biological activity [129,130,131,132,133,134,135,136,137]. Typically, the shape and electron density of boundary orbitals indicate the most reactive regions of a molecule. However, in other cases, compounds with the same biological activity follow a similar pattern of boundary orbitals [48]. The frontier molecular orbitals for the design structures were retrieved from the optimized output using the B3LYP/6-31G(d,p) level of theory; the isosurfaces were generated using 0.02 eV isovalues.

### 3.10. Chemistry

Reagents and solvents were purchased from Sigma (St. Louis, MO, USA) and Merck (Darmstadt, Germany). Melting points were measured using a Stuart SMP3 melting point apparatus. IR spectra were recorded on a Shimadzu FTIR 8400 ATR spectrophotometer. Mass spectra were run on a SHIMADZU-GCMS 2010-DI-2010 spectrometer (equipped with a direct inlet probe) operating at 70 eV. In addition, ^1^H and ^13^C NMR spectra were recorded on a Bruker Avance 400 spectrophotometer operating at 400 MHz and 100 MHz, respectively, using DMSO-d_6_ as solvent and tetramethylsilane as the internal standard. TLC analyses were performed on 0.2 mm pre-coated aluminum plates of silica gel 60 F_254_ (Merck, Darmstadt, Germany) and spots visualized were with ultraviolet irradiation.

**General procedure for the synthesis of the (*E*)-3-(3-oxobut-1-en-1-yl)quinolin-2(1H)-ones 16a**–**e.** A mixture of acetone (10 mL, 136 mmol), the corresponding 3-formyl-2-oxo-quinoline precursors **15a**–**e** (1.0 mmol) and 20% aq NaOH (5 mL) was stirred at room temperature for 15 min and the evolution of the reaction was checked by TLC. Once the reaction was complete, the resulting precipitate was collected by filtration under vacuum and washed several times with water and ethanol to afford **16a**–**e**. No further purification was required.

**General procedure for the synthesis of the novel quinolinone-based thiosemicarbazones 11a**–**e.** A mixture of (*E*)-3-(3-oxobut-1-en-1-yl)quinolin-2(1H)-one **16a**–**e** (1 mmol), thiosemicarbazide (1.5 mmol), and acetic acid (50 µL) in EtOH (10 mL) was heated at reflux for 6–8 h. The precipitate thus produced was filtered and washed several times from EtOH to give products **11a**–**e**. No further purification was required.

**(*E*)-2-((*E*)-4-(6-Methyl-2-oxo-1,2-dihydroquinolin-3-yl)but-3-en-2-ylidene)hydrazine-1-carbothioamide 11a**. Yellow solid. 80% yield; mp = 240 °C. FTIR (ATR) ν (cm^−1^): 3404 (NH), 3095 (NH), 1600 (C=O). ^1^H NMR (400 MHz, DMSO-d_6_) δ 2.12 (s, 3H, CH_3_), 7.10 (d, J = 16.5 Hz, 1H, =CH), 7.14 (m, 1H), 7.28 (d, J = 8.2 Hz, 1H), 7.42 (d, J = 16.5 Hz, 1H, =CH), 7.5 (m, 1H,) 7.67 (d, 1H), 7.8 (s, 1H, NH_2_), 8.15 (s, 1H), 8.30 (s, 1H, NH_2_), 10.30 (s, 1H, NH), 12.90 (s, 1H, NH). ^13^C NMR (101 MHz, DMSO-d_6_) δ 12.4 (CH_3_), 115.3, 119.8 (Cq), 122.6, 127.9, 128.5, 129.1, 130.9(Cq), 137.1, 138.5, 149.5 (Cq), 161.2 (C=O), 179.1 (Cq). EI MS (70 eV): *m*/*z* (%): 286 (M^+^, 10), 170 (100), 115 (61), 211 (42), 140 (40), 60 (41), 226 (38), 89 (36), 152 (30), 269 (27), 197 (22), 244 (21).

**(*E*)-2-((*E*)-4-(6-Methyl-2-oxo-1,2-dihydroquinolin-3-yl)but-3-en-2-ylidene)hydrazine-1-carbothioamide 11b**. Yellow solid. 70% yield; m.p = 251 °C. FTIR (ATR) ν (cm^−1^): 3415 (NH), 3245 (NH), 3155 (NH), 1599 (C=O). ^1^H NMR (400 MHz, DMSO-d_6_) δ 2.14 (s, 3H, CH_3_), 2.35 (s, 3H, CH_3_), 7.13 (d, J = 16.5 Hz, 1H, =CH), 7.22 (d, J = 8.3 Hz, 1H), 7.33 (d, J = 9.8 Hz, 1H), 7.49–7.42 (m, 2H), 7.8 (s, 1H, NH), 8.08 (s, 1H,), 8.28 (s, 1H, NH), 10.28 (s, 1H, NH), 11.90 (s, 1H, NH). ^13^C NMR (101 MHz, DMSO-d_6_) δ 12.4 (CH_3_), 20.9 (CH_3_), 115.2, 119.8 (Cq), 127.8, 127.9, 129.4, 131.5(Cq), 132.1, 132.3, 136.5 (Cq), 137.0, 149.7 (Cq), 161.2 (C=O), 179.2 (Cq). EI MS (70 eV): *m*/*z* (%): 300 (M^+^, 7), 184 (100), 60 (67), 42 (44), 225 (39), 40 (38), 283 (37), 128 (37), 154 (36), 77 (33), 240 (32), 115 (31), 211 (22), 167 (19), 258 (15), 102 (15).

**(*E*)-2-((*E*)-4-(8-Methyl-2-oxo-1,2-dihydroquinolin-3-yl)but-3-en-2-ylidene)hydrazine-1-carbothioamide 11c.** Yellow solid. 75% yield; mp = 241 °C. FTIR (ATR) ν (cm^−1^): 3413 (NH), 3228 (NH), 3149 (NH), 1581 (C=O). ^1^H NMR (400 MHz, DMSO-d_6_) δ, 2.16 (s, 3H, CH_3_). 2.45 (s, 3H, CH_3_), 7.19–7.08 (m, 2H), 7.35 (d, J = 7.2 Hz, 1H), 7.43 (d, J = 16.6 Hz, 1H, =CH), 7.53 (d, J = 7.8 Hz, 1H), 7.83 (s, 1H, NH), 8.17 (s, 1H), 8.29 (s, 1H, NH), 10.30 (s, 1H, NH), 11.08 (s, 1H, NH). ^13^C NMR (101 MHz, DMSO-d_6_) δ 12.4 (CH_3_), 17.6 (CH_3_), 119.9 (Cq), 122.4, 123.7 (Cq), 126.7, 127.6 (Cq), 128.9, 132.1, 132.6 (Cq), 136.9 (Cq), 137.6, 149.6 (C=N, Cq), 161.8 (C=O, Cq), 179.22 (Cq). EI MS (70 eV): *m*/*z* (%): 300.05 (M^+^, 13), 184 (100), 60 (53), 240 (45), 225 (41), 154 (32), 283 (32), 128 (30), 141 (27), 115 (26), 258 (26), 77 (26), 211 (24), 167 (18), 102 (13).

**(*E*)-2-((*E*)-4-(7-Chloro-2-oxo-1,2-dihydroquinolin-3-yl)but-3-en-2-ylidene)hydrazine-1-carbothioamide 11d**. Yellow solid. 85 % yield; mp = 246 °C. FTIR (ATR) ν (cm^−1^): 3465 (NH), 3320 (NH), 3162 (NH), 1594 (C=O). ^1^H NMR (400 MHz, DMSO-d_6_) δ 2.14 (s, 3H, CH_3_), 7.10 (d, J = 16.6 Hz, 1H, =CH), 7.24 (dd J = 8.4 Hz, 1H), 7.33 (s, 1H), 7.44 (d, J = 16.5 Hz, 1H, =CH), 7.70 (dd, 1H), 7.80 (s, 1H, NH), 8.17 (s, 1H), 8.29 (s, 1H, NH), 10.31 (s, 1H, NH), 12.05 (s, 1H, NH). ^13^C NMR (101 MHz, DMSO-d_6_) δ 12.4 (CH_3_), 114.6, 118.6 (Cq), 122.8, 128.3, 128.8 (Cq), 130.3, 132.7, 135.2 (Cq), 136.4, 139.2 (Cq), 149.4 (Cq), 161.2 (C=O), 179.2 (Cq). EI MS (70 eV): *m*/*z* (%): 320.05 (M^+^, 2), 303 (74), 204 (100), 140 (76), 77 (77), 140 (75), 303 (72), 71 (64), 69 (68), 97 (55), 43 (54), 261 (49), 57 (47), 94 (48), 245 (44), 113 (43), 151 (41), 208 (39), 305 (29), 313 (25), 263 (25), 167 (20), 230 (20), 186 (15), 288 (14).

**(*E*)-2-((*E*)-4-(6-Bromo-2-oxo-1,2-dihydroquinolin-3-yl)but-3-en-2-ylidene)hydrazine-1-carbothioamide 11e**. Yellow solid. 85% yield; mp = 253 °C. FTIR (ATR) ν (cm^−1^): 3412 (NH), 3267 (NH), 3162 (NH), 1646 (C=O). ^1^H NMR (400 MHz, DMSO- *d_6_*) δ 2.14 (s, 3H, CH_3_), 7.10 (d, *J* = 16.5 Hz, 1H, =CH), 7.25 (d, *J* = 8.7 Hz, 1H), 7.44 (d, *J* = 16.6, 1.8 Hz, 1H, =CH), 7.62 (d,d, *J* = 8.7, 2.1 Hz, 1H), 7.81 (s, 1H, NH_2_), 7.89 (d, *J* = 1.8 Hz, 2H), 8.11 (s, 1H), 8.30 (s, 1H, NH), 10.31 (s, 1H, NH), 12.09 (s, 1H, NH). ^13^C NMR (101 MHz, DMSO- *d_6_*) δ 12.4. (CH_3_), 114.1 (Cq), 117.4, 121.5 (Cq), 128.7, 129.1 (Cq), 130.2, 133.0, 133.3, 135.7, 137.4 (Cq), 149.4 (Cq), 161.1 (Cq), 179.2 (Cq). EI MS (70 eV): *m/z* (%): 140 (100), 365.85 (M^+^, 2), 247 (81), 249 (80), 114 (61), 289 (50), 143 (52), 348 (45), 346 (43), 169 (35), 304 (33), 36 (34), 42 (34), 274 (32), 89 (22), 276 (22), 99 (17), 151 (18), 210 (12), 319 (10).

### 3.11. Antimycobacterial Activity

Antimicrobial activity, was measured by spot culture growth inhibition assay (SPOTi) [138]. In order to evaluate the minimum inhibitory concentration (MIC) values for the different synthetic compounds **11**, these were tested against the laboratory strains: *M. tuberculosis H37Rv*, *M. tuberculosis lineage orphan*, *M. tuberculosis lineage Beijing*, *M. tuberculosis lineage LAM 9*, *M. tuberculosis lineage Haarlem*, *M. tuberculosis ATCC 35838 (rifampicin resistant)* and *M. tuberculosis ATCC 35822* (isoniazid resistant). Biological activity was tested in a level 3 biosafety laboratory of the Colombian National Health Institute. *M. bovis* assays were performed at Universidad del Norte at the biological and chemical research laboratory. Briefly, the compound was dissolved in dimethylsulfoxide (DMSO) in a 200 mg/mL stock solution; from this, a serial dilution was made in a sterile 24-well microplate, allowing for evaluation concentrations of 100–0.01 mg/L. First, 2.0 µL of the dilutions were dispensed in each dish. Next, 2.0 mL of Middlebrook 7H10 medium was added appropriately and supplemented with glycerol and OADC (oleic acid, albumin, dextrose, and catalase). The medium was allowed to cool, and then in the middle of each well 2.0 µL of a dilution of the inoculum (containing about 10^6^ CFU/mL) of *M. tuberculosis* H37Rv or *M. bovis* BCG was added. The isoniazid drug was used as a positive standard. The plates were covered for 2–3 weeks at 37 °C. After the incubation period, the plates were observed and the MIC was determined as the minimum concentration on which growth was not observed. The experiment was repeated on a different day observing exactly the same results.

### 3.12. Cytotoxicity Assay

Vero cells (ATCC^®^ CCL-81 ™), were cultured in Dulbecco Modified Eagle Medium (DMEM) supplemented with 10% bovine fetal serum and 1% streptomycin–penicillin and passaged twice before the assay in 21 cm^2^ cell culture Petri dishes at 37 °C in 5% CO_2_ incubator and 100% humidity. Cells were then cultured in 96-well plates for 24 h before the assay to a cell density of 104 cells per well. A 96-well master plate was used to dilute of the compounds in DMEM medium, by diluting 5 mL of the 100 mg/mL DMSO stock of the compounds into 195 mL of the DMEM medium and performing twofold serial dilution with 100 mL. One hundred microliters of this solution was transferred to each plate containing the Vero cells and incubated for 48 h. Five percent NaDS was used as a positive control and DMSO as negative control under the same dilution conditions. Ten milliliters of a freshly prepared MTT (3-(4,5-dimethyl-2-thiazolyl)-2,5-diphenyl-2H-tetrazoliumbromide) solution in PBS at 5 mg/mL was added to each well, and the plates were further incubated for 1.5 h. The media was then removed and 130 mL of DMSO was added to each well. After 30 min of incubation, the absorbance was read at 530 nm on a microplate reader. The experiment was performed in duplicate on different days with different cell cultures and different stock of the compounds. The IC_50_ (in µM) values were determined by interpolation from the mean absorbance data of 100% viability (negative control) and 0% viability (positive control).

## 4. Conclusions

Thirty-two compounds with anti-TB activity against the *M. tuberculosis* H37RV strain were retrieved from the literature to obtain possible quantitative structure–anti-TB activity correlations. These compounds were optimized at the B3LYP/6-31G (d,p) level of theory; the minimum energy structures were used to calculate electronic, thermodynamic, and topographical molecular descriptors. As a result, model 3 was chosen according to statistics and the lowest number of attributes, so it was used as a guiding model for generating new compounds. The van der Waals volume, electron density, and electronegativity model results suggest a pivotal role in anti-TB activity; as such, van der Waals volume increases biological activity and electron density and the electronegativity decrease the anti-TB activity. Five new compounds were designed by taking compound 26 as a base structure and adding substituents according to model 3. The designed compounds were compared electronically with reported anti-TB compounds, such as BTZ043 and isoniazid. It was found that compounds with frontier orbitals distributed on opposite sides of the molecules exhibited higher activity values than those without this behavior. In addition, docked compounds against enoyl-acyl carrier protein reductase (InhA) and decaprenylphosphoryl-β-D-ribose-2’-oxidase (DprE1) showed affinity values ranging from −8.5 to −7.1 Kcal/mol; compounds **11d** and **11e** showed the best behavior towards both molecular targets. Protein–compound molecular dynamics calculations resulted in negative binding energy in the range of −71.3 to −12.7 kcal/mol. In all cases, the compounds bound to each target at the catalytic site reported in the literature, which means that there is a high probability that they were inhibitors of these two proteins. The newly designed compounds were synthesized and evaluated for their anti-TB activity against *M. bovis BCG*, *M. tuberculosis* H37Rv, and six strains of *M. tuberculosis* resistant to rifampicin and isoniazid. Most compounds showed better MIC values than the reference drugs (isoniazid and oxafloxacin). Compounds **11d** and **11e,** with chlorine and bromine groups on the quinolinone ring, were found to be the most active with MIC values of 0.15 µM and 0.13 µM, respectively, against *M. tuberculosis* H37Rv and selectivity index of >10. This study suggests that **11d** and **11e** possess great potential in developing of new anti-TB drugs and can serve as hits for in vivo studies to validate their potential as lead compounds.

## Figures and Tables

**Table 1:** Pearson correlations obtained for model 3.

Attribute	8a	24a	χ
**8a**	1	0.02	−0.51
**24a**	0.02	1	0.25
**χ**	−0.51	0.25	1

**Table 2:** Different statistical values of the predictive capacity of QSAR model 3.

Size	r^2^	Q_LOO_	Q_LMO_		Q_ext_	Q_boot_	a (R^2^)	a (Q^2^)	F
3	0.83	0.78	0.72		0.81	0.716	0.13	−0.27	47.96
		**pMIC** = 6.401 + 2.877(**8a**) − 0.228(**24a**) − 19.793**χ**							

**Table 3:** Validation based on the Tropsha’s test for QSAR model 3.

	Leave-One-Out Validation		External Validation	
Criterion	Result	Assessment	Result	Experimental
r^2^ > 0.6	0.83	PASS	0.83	PASS
r^2^_val_ > 0.5	0.78	PASS	0.81	PASS
(r^2^_val_ − r^2^_0_)/r^2^_val_ < 0.1	0.00	PASS	0.00	PASS
(r^2^_val_ − r^2^_0_)/r^2^_val_ < 0.1	0.06	PASS	0.00	PASS
abs (r^2^_0_ − r′^2^_0_) < 0.1	0.05	PASS	0.00	PASS
0.85 < *K* < 1.15	0.99	PASS	0.99	PASS
0.85 < *K*′ < 1.15	0.99	PASS	1.00	PASS

**Table 4:** Molecular descriptor values and minimum inhibitory concentration predicted for five compounds designed using the QSAR model obtained herein.

Comp.	R	Y_p_	MIC_p_ (µM)	8a	24a	χ	HD	HA	P	MW
**11a**	H	5.49	0.23	0.25	−2.29	0.10	3	3	−0.12	286.35
**11b**	6-Me	5.56	0.22	0.23	−3.02	0.11	3	3	0.97	300.38
**11c**	8-Me	5.93	0.10	0.54	−0.93	0.11	3	3	0.97	300.38
**11d**	7-Cl	6.82	0.14	0.57	−4.26	0.11	3	3	1.34	320.80
**11e**	6-Br	7.03	0.09	0.57	−4.99	0.10	3	3	1.49	365.25

**8a** = (SD_B_AB_nCi_2_SS8_P_LGP [2,3,4] v-c_MAS); **24a** = (K_B_AB_nCi_2_NS4_P_KA_e-c_MAS); **χ** = electronegativity; **HD** = donor H; **HA** = acceptor H; **P** = coefficient partition; **MW** = molecular weight.

**Table 5:** Vina scores were obtained for the designed compounds and the reported target inhibitors.

Compounds	Vina Score (kcal/mol)	RMS		RMS
	DPrE1		InhA	
**11a**	−7.30	1.8	−7.60	2.3
**11b**	−7.10	2.4	−8.20	1.8
**11c**	−7.50	1.6	−8.40	1.5
**11d**	−7.20	2.0	−8.40	1.3
**11e**	−7.90	1.5	−8.50	1.2
**BTZ043**	−10.70	1.3	−8.30	2.4
**INH-NADP complex**	−6.6	1.5	−10.40	0.8

**Table 6:** Free binding energy of the interaction in the complex between studied ligands and enzymes.

Enzyme	Compound	van der Waals (kcal/mol)	Electrostatic (kcal/mol)	SASA (kcal/mol)	Binding Energy (kcal/mol)
InhA	**11c**	−38.20	−7.40	−4.30	−19.30
	**11d**	−36.60	−77.20	−4.60	−71.30
	**11e**	−45.0	−7.30	−4.20	−22.90
DprE1	**11c**	−40.00	−14.20	−4.00	−13.60
	**11d**	−46.70	−19.60	−4.60	−12.70
	**11e**	−38.20	−13.10	−3.90	−14.80

**Table 7:** General approach for the preparation of the target quinolinone-thiosemicarbazone hybrids **11a**–**e**.

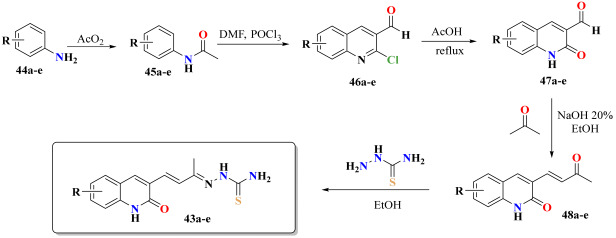			
Compound	R	Yield	Time (h)
**11a**	H	80	6
**11b**	6-CH_3_	70	6
**11c**	8-CH_3_	75	6
**11d**	7-Cl	85	8
**11e**	6-Br	85	8

**Table 8:** Antimycobacterial activity against *M. tuberculosis* H37Rv and *M. bovis.*

Compound	*M. bovis* BCG	*M. tuberculosis* H37Rv
	MICs in µM	
**11a**	0.17	0.17
**11b**	0.16	0.16
**11c**	0.33	0.16
**11d**	0.03	0.15
**11e**	0.13	0.13
**Isoniazid**	0.36	0.36

**Table 9:** Antimycobacterial activity against *M. tuberculosis*.

Comp.	*M. tuberculosis Orphan*	*M. tuberculosis Beijing*	*M. tuberculosis LAM 9*	*M. tuberculosis Haarlem*	*M. tuberculosis* ATCC 35838	*M. tuberculosis* ATCC 35822
	MICs in µM					
**11a**	3.49	1.75	0.35	17.46	3.49	3.49
**11b**	33.29	1.66	16.65	3.33	3.33	3.33
**11c**	66.58	0.33	3.29	33.29	1.66	0.33
**11d**	6.23	0.33	3.12	1.56	3.33	6.23
**11e**	27.38	0.27	2.74	27.37	2.73	1.37
**Oxafloxacin**	2.67	2.76	2.76	2.76	2.76	2.76

**Table 10:** Cytotoxic activity (IC_50_) in Vero cells and selectivity index (SI) of the active synthetic hybrids **11a**–**e** strains of *M. tuberculosis*.

Compound	Cytotoxicity IC_50_-Vero Cells µM	a	b	c	d	e	f	g	h
	Selectivity Index (SI) (SI = IC_50_/MIC)								
**11a**	1.91	11.24	11.24	0.55	1.09	5.46	0.11	0.55	0.55
**11b**	3.53	22.06	22.06	0.11	2.13	0.21	1.06	1.06	1.06
**11c**	1.90	5.75	11.88	0.03	5.76	0.58	0.06	1.14	5.76
**11d**	1.65	55.00	11.00	0.26	5.00	0.53	1.06	0.50	0.26
**11e**	2.20	16.92	16.92	0.08	8.15	0.80	0.08	0.81	1.61

(**a**) *M. bovis* BCG; (**b**) *M. tuberculosis* H37Rv; (**c**) *M. tuberculosis orphan*; (**d**) *M. tuberculosis Beijing*; (**e**) *M. tuberculosis LAM 9* (sit 42); (**f**) *M. tuberculosis Haarlem*; (**g**) *M. tuberculosis* ATCC 35838 RR; (**h**) *M. tuberculosis* ATCC 35822 RI.

**Table 11:** Docking information.

Protein	PDB:ID	Active Site (x, y, z)	Inhibitor	Grilla Box Size (x, y, z)
DprE1	6HFW	13.869, −21.448, 37.131	8-(oxidanylamino)-2-piperidin-1-yl-6-(trifluoromethyl)-1,3-benzothiazin-4-one (BTZ043)	22, 22, 22
InhA	2PR2	−1.702, −27.226, 15.656	INH-NADP	22, 22, 22

## Data Availability

Not applicable.

## Supplementary Materials

The following are available online at https://www.mdpi.com/article/10.3390/antibiotics12010061/s1, Figure S1: (a) Parity diagram MIC exp versus MICp; (b) Parity diagram QLoo vs. MIC exp; (c) graphic residual vs. MIC exp; (d) graphic residual QLooc vs MIC exp, Figure S2. The frontier orbital for the designed compounds 11a–e from the QSAR model. HOMO: Highest occupied molecular orbital; LUMO: low unoccupied molecular orbital. BTZ: Benzothiazinone derivatives, Figure S3. ^1^H and ^13^C NMR spectrum of **11a**, Figure S4. ^1^H, ^13^C NMR and DEPT 135 NMR spectrum of **11b**, Figure S5. ^1^H, ^13^C NMR and DEPT 135 NMR spectrum of **11c**, Figure S6. ^1^H, ^13^C NMR and DEPT 135 NMR spectrum of **11d**, Figure S7. ^1^H, ^13^C NMR and DEPT 135 NMR spectrum of **11e**, Table S1: Observed and predictive and experimental activities (and their residuals) of the set of quinoline derivatives.
