# Supplementary material for: QSAR Studies, Molecular Docking, Molecular Dynamics, Synthesis, and Biological Evaluation of Novel Quinolinone-Based Thiosemicarbazones against Mycobacterium tuberculosis
Source: Antibiotics (Basel). 2022 Dec 29;12(1):61. doi: 10.3390/antibiotics12010061 (PMC9854539; doi:10.3390/antibiotics12010061)
Supplement: Supplementary file 1 [file antibiotics-12-00061-s001.zip › antibiotics-2013201-supplementary.pdf]

Supplementary Materials

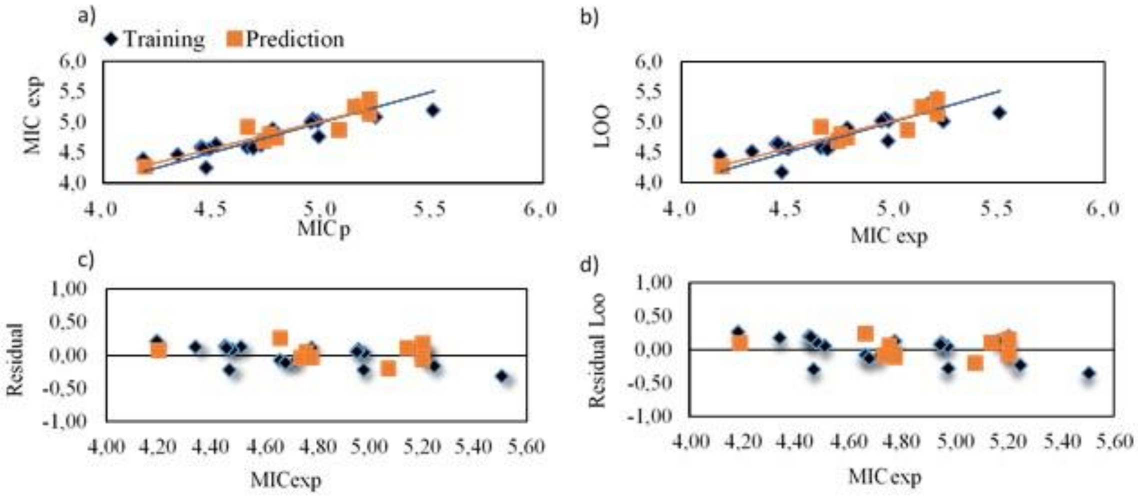

Figure S1. a) Parity diagram MIC exp versus MICp; b) Parity diagram QLoo vs MIC exp; c) graphic residual vs MIC exp; d) graphic residual QLooc vs MIC exp.

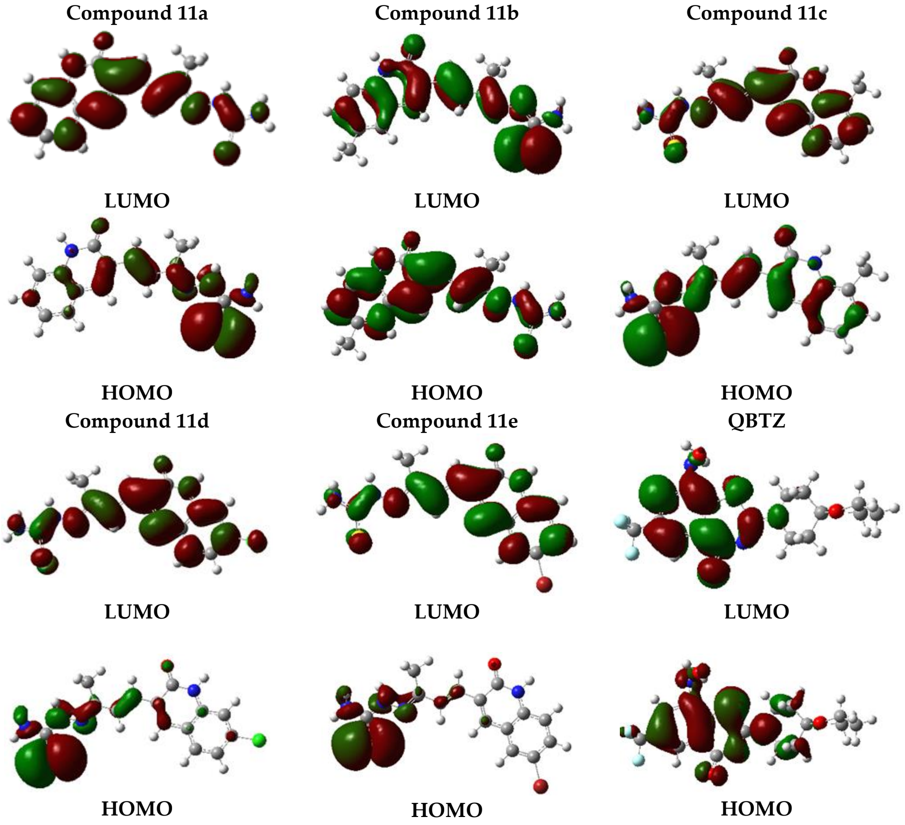

Figure S2. The frontier orbital for the designed compounds 11a-e from the QSAR model. HOMO: Highest occupied molecular orbital; LUMO: low unoccupied molecular orbital. BTZ: Benzothiazinone derivatives.

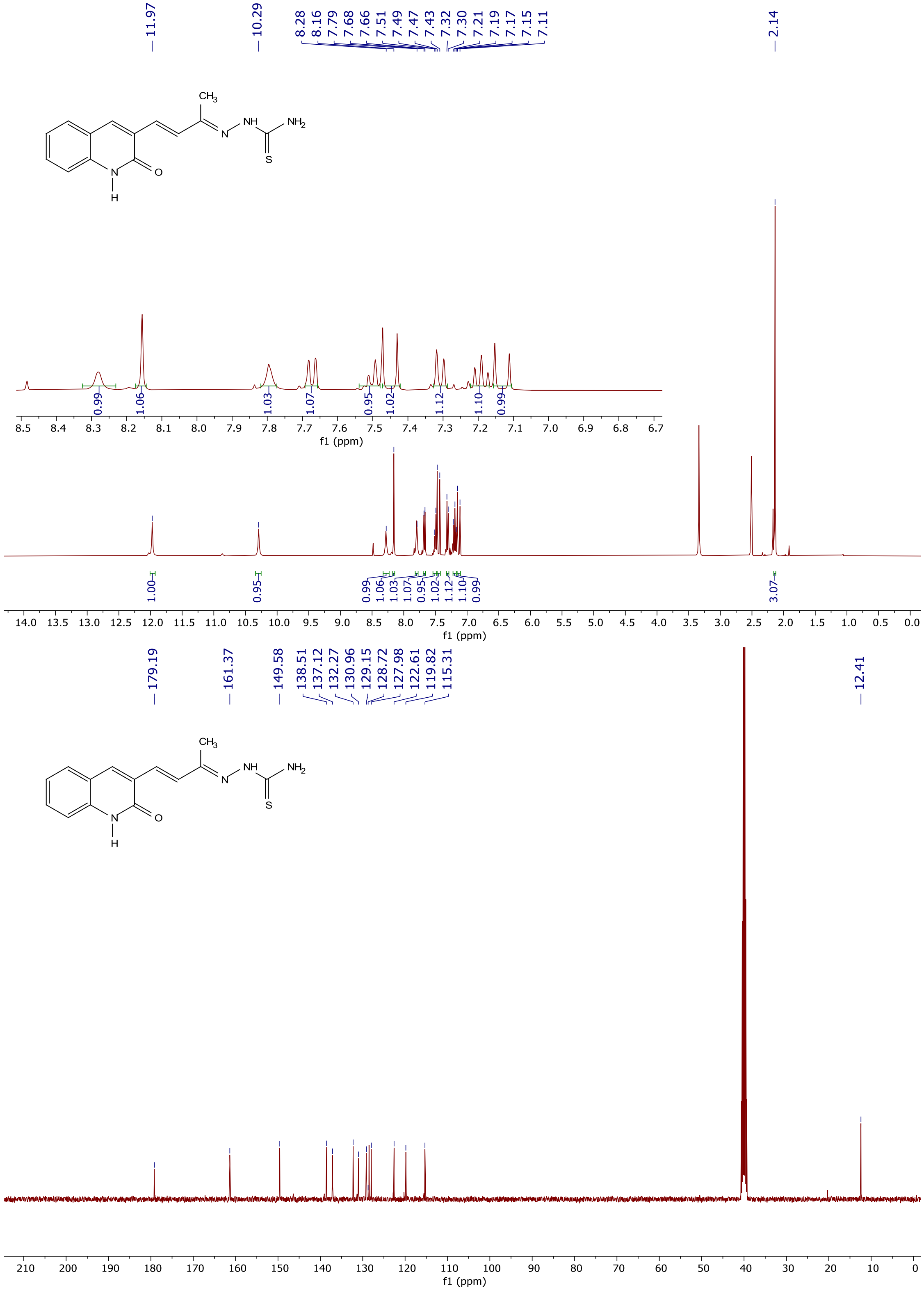

Figure S3. ^{1}H and ^{13}C NMR spectrum of **11a**

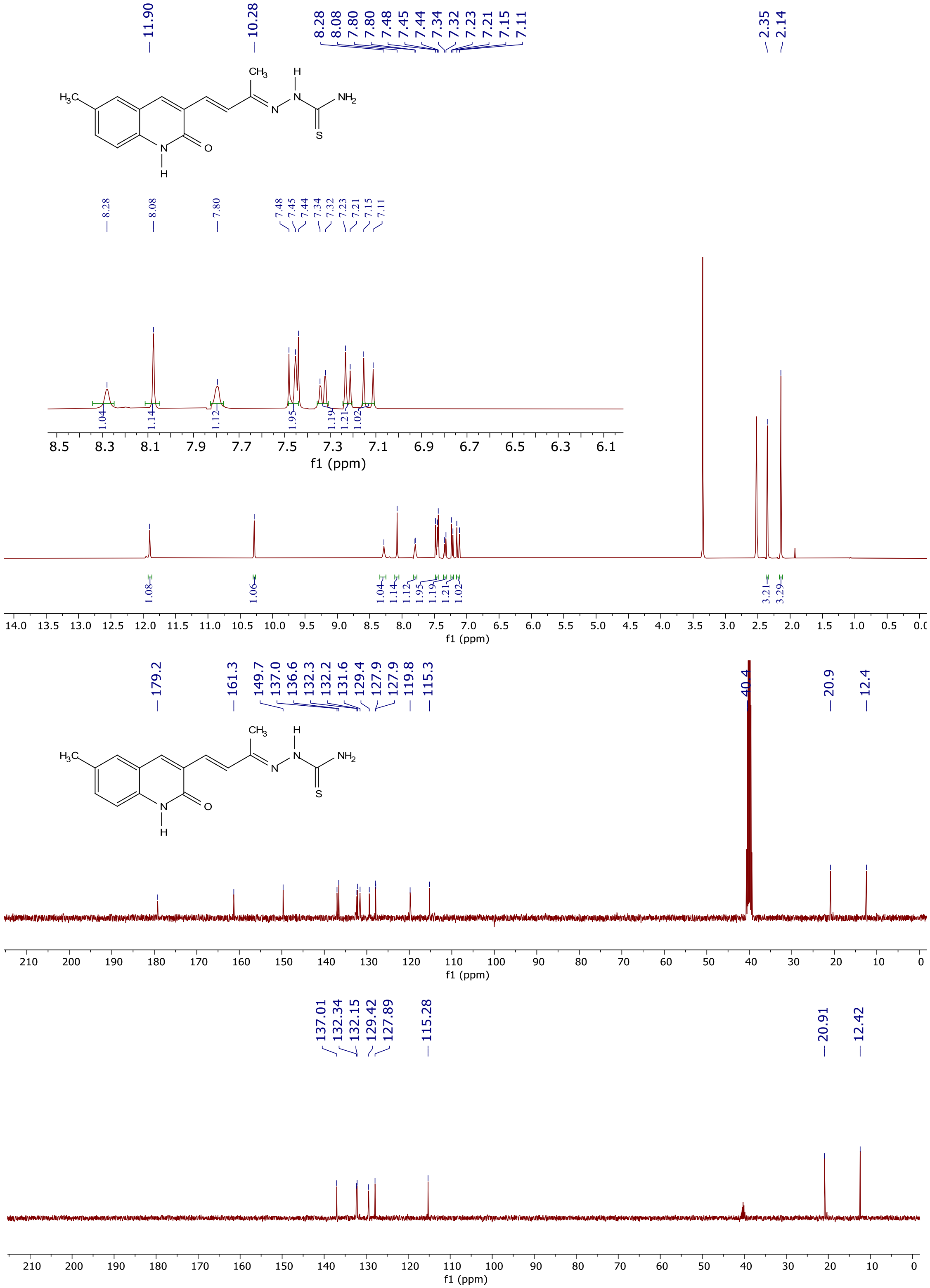

Figure S4. ^{1}H, ^{13}C NMR and DEPT 135 NMR spectrum of **11b**

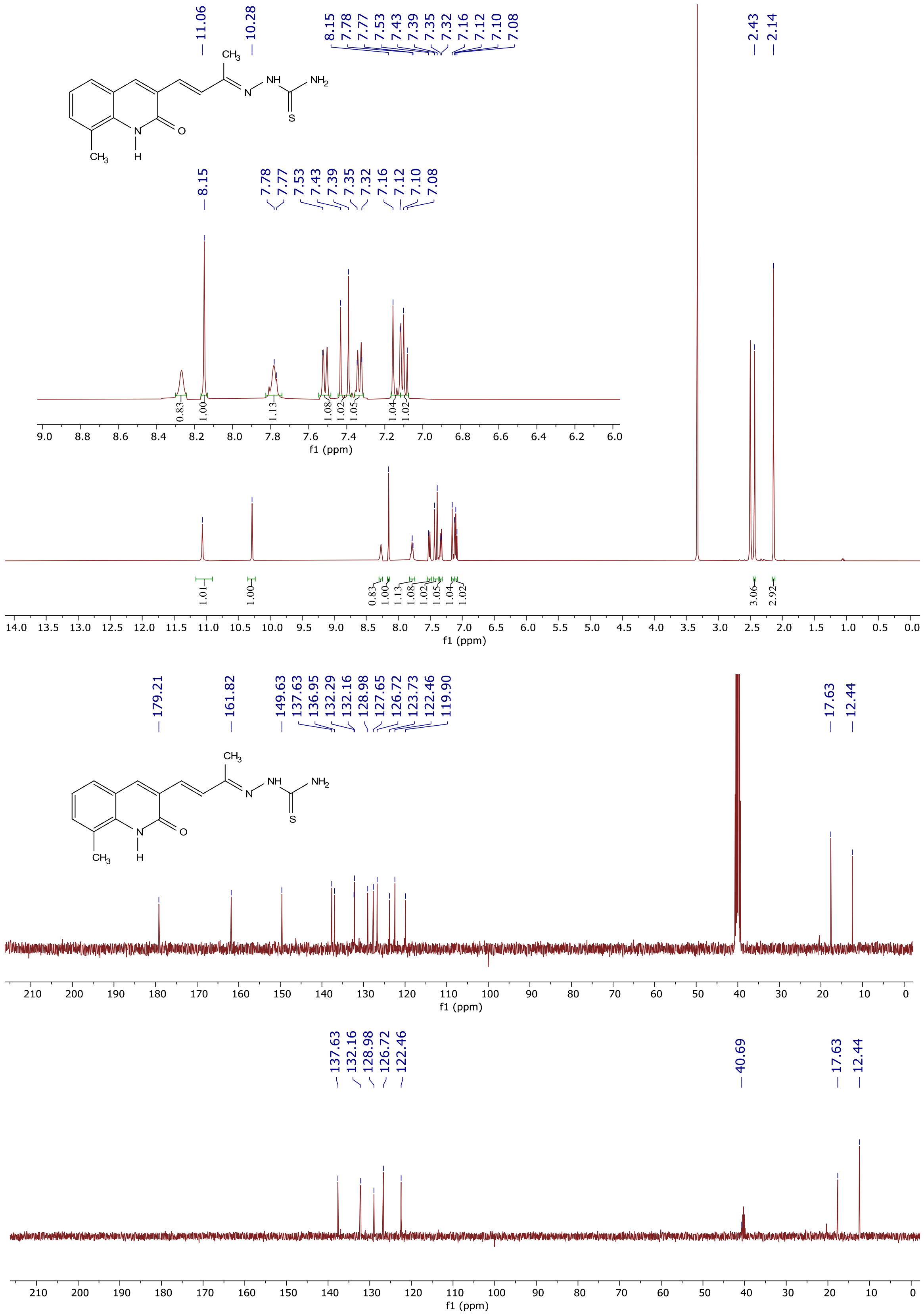

Figure S5. ^{1}H, ^{13}C NMR and DEPT 135 NMR spectrum of **11c**

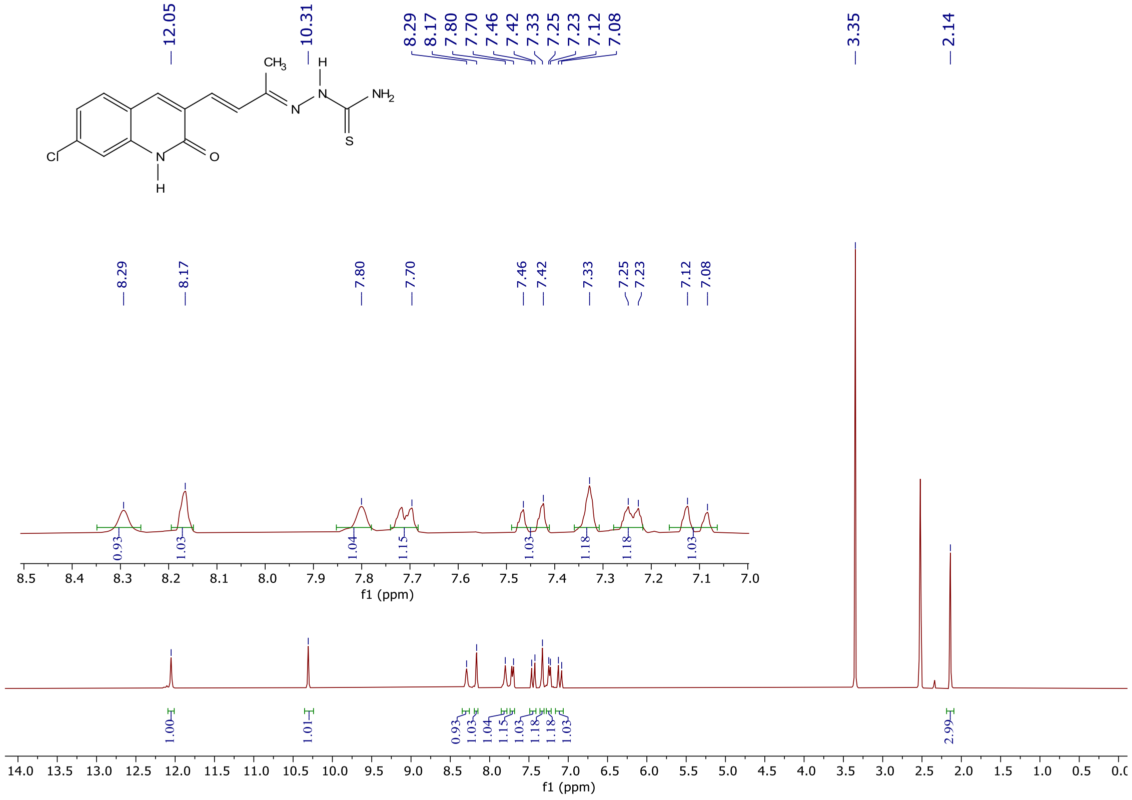

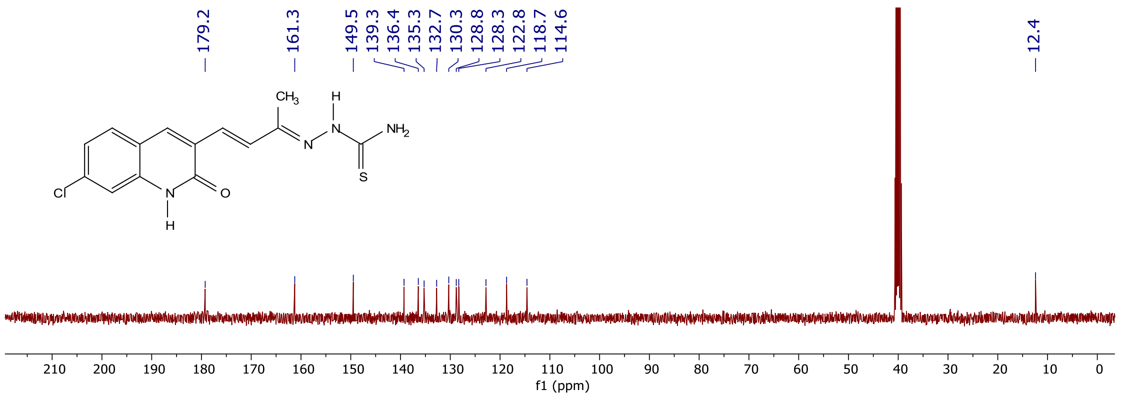

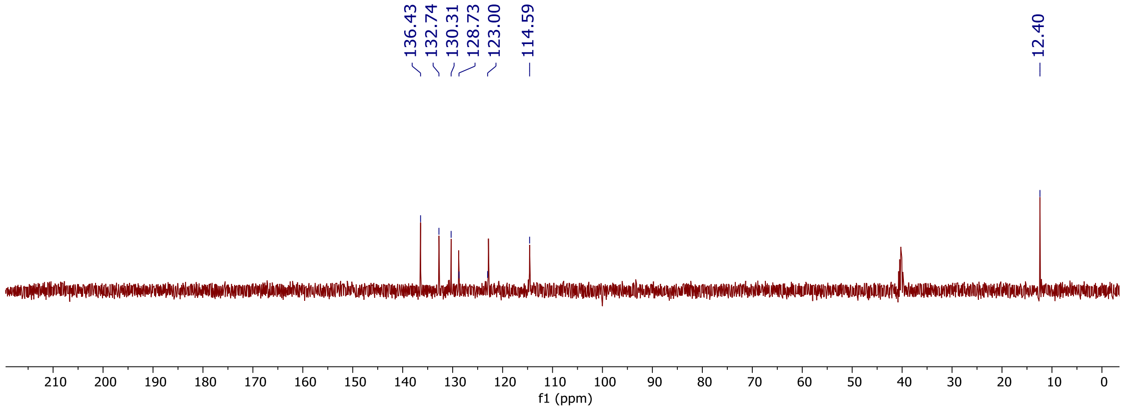

Figure S6. ^{1}H, ^{13}C NMR and DEPT 135 NMR spectrum of **11d**

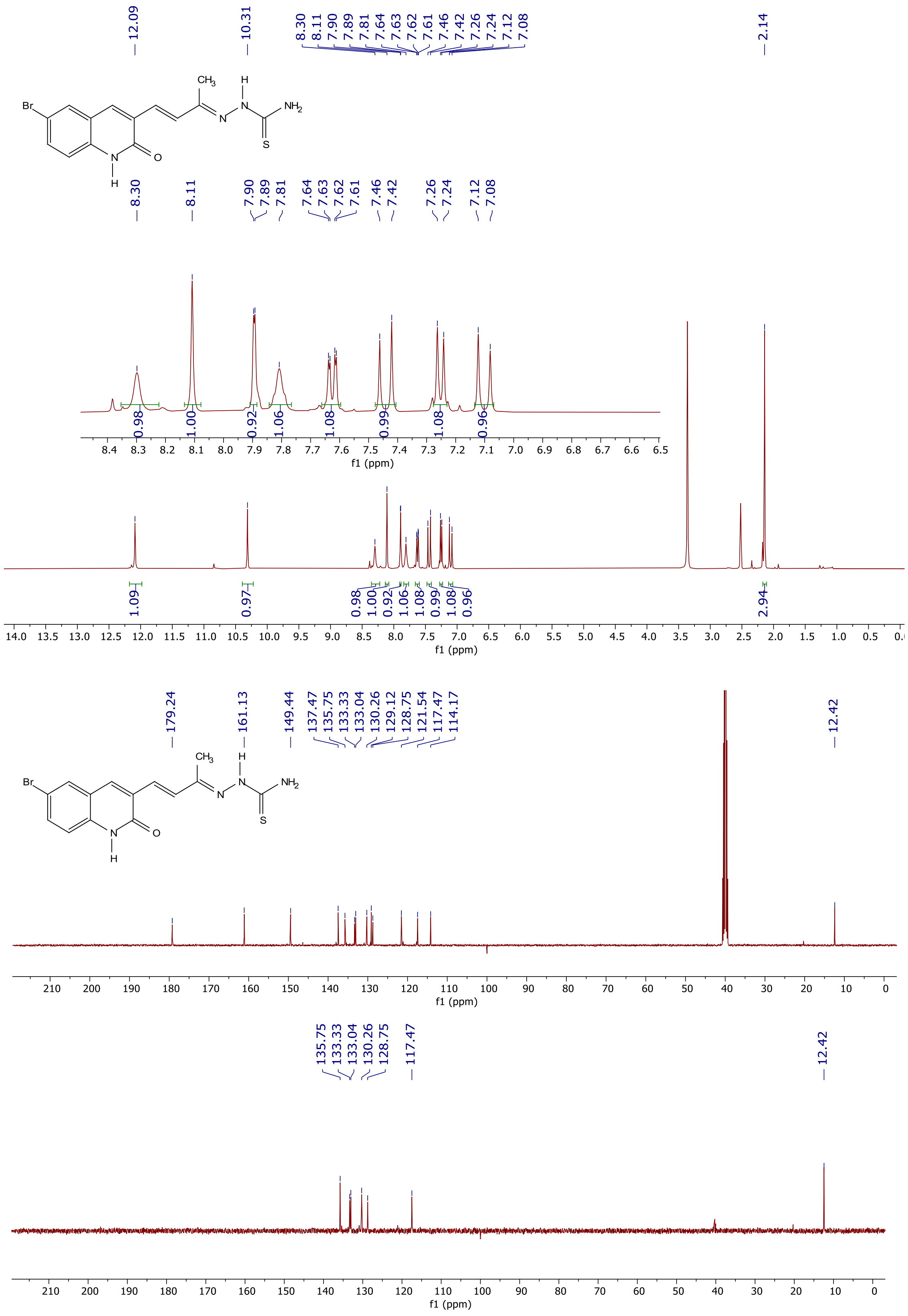

Figure S7. ^{1}H, ^{13}C NMR and DEPT 135 NMR spectrum of **11e**

Table S1. Observed and predictive and experimental activities (and their residuals) of the set of quinoline derivatives

Compound	MIC(μM)	MIC exp (log (1/molarMIC))	MIC_p	Residual	Q_{Loo}	Residual Q_{Loo}
17	6.89	5.16	5.28	0.12	5.30	0.14
18	10.65	4.97	5.02	0.04	5.02	0.05
19*	7.26	5.13	5.25	0.11	5.25	0.11
20	6.25	5.2	5.43	-0.06	5.13	-0.07
21*	6.25	5.2	5.14	-0.06	5.13	-0.07
22	6.25	5.20	5.18	-0.03	5.17	-0.03
23	6.25	5.20	5.28	0.08	5.29	0.09
24*	6.25	5.20	5.38	0.18	5.36	0.16
25	6.25	5.20	5.33	0.13	5.40	0.20
26	3.12	5.51	5.20	-0.31	5.16	-0.35
27	19.33	4.71	4.62	-0.09	4.61	-0.10
28	10.57	4.98	4.76	-0.22	4.69	-0.28
29	45.73	4.34	4.46	0.12	4.52	0.18
30	21.75	4.66	4.59	-0.07	4.58	-0.08
31	20.74	4.68	4.57	-0.11	4.56	-0.13
32	35.77	4.45	4.59	0.14	4.64	0.20
33	11.11	4.95	5.05	0.09	5.06	0.11
34*	21.75	4.66	4.92	0.26	4.90	0.24
35*	8.48	5.07	4.88	-0.20	4.87	-0.20
36	16.76	4.78	4.89	0.12	4.91	0.13
37*	17.54	4.76	4.80	0.05	4.82	0.06
38	30.92	4.51	4.64	0.13	4.65	0.14
39	33.53	4.47	4.56	0.08	4.57	0.09
40	35.07	4.46	4.57	0.12	4.60	0.14
41	14.98	4.82	4.66	-0.16	4.65	-0.18
42	65.37	4.18	4.40	0.21	4.45	0.27

**43***	64.42	4.19	4.27	0.08	4.29	0.10
**44**	33.75	4.47	4.25	-0.22	4.18	-0.29
**39***	18.42	4.73	4.69	-0.04	4.65	-0.08
**40***	16.72	4.78	4.74	-0.03	4.65	-0.12
**41**	5.73	5.24	5.08	-0.16	5.01	-0.23
**42**	11.34	4.95	5.00	0.06	5.02	0.08

Denotes the prediction; Residual= MICp-MICexp; Residual Q_{Loo}=Q_{loo}-MICexp
